# In silico-driven protocol for hit-to-lead optimization: a case study on PDE9A inhibitors

**DOI:** 10.1007/s10822-025-00729-7

**Published:** 2025-12-19

**Authors:** Hiroyuki Ogawa, Masateru Ohta, Mitsunori Ikeguchi

**Affiliations:** 1 Graduate School of Medical Life Science, Yokohama City University, 1-7-29 Suehiro-cho, Tsurumi-ku, Yokohama 230-0045 Japan; 2 Central Pharmaceutical Research Institute, Japan Tobacco Inc., 1-1, Murasaki-cho, Takatsuki, Osaka 569-1125 Japan; 3 HPC- and AI-driven Drug Development Platform Division, Center for Computational Science, RIKEN 1-7-22, Suehiro-cho, Tsurumi-ku, Yokohama 230-0045 Japan

**Keywords:** Non-equilibrium switching, Hit-to-lead, Drug discovery, PDE9A

## Abstract

Hit-to-lead (H2L) optimization is a critical stage in small-molecule drug discovery, where efficient exploration of chemical space is required to identify promising lead compounds. Conventional H2L workflows rely on iterative synthesis and experimental evaluation, which limit the range of chemical space that can be explored. In contrast, in silico approaches enable efficient selection of promising compounds from a much larger chemical space by generating large numbers of virtual compounds and evaluating them computationally. To harness this potential, we developed an in silico–driven H2L protocol that integrates molecular generation, binding affinity prediction based on relative binding free energies calculated using the non-equilibrium switching (NES) method, and the evaluation of key properties—such as solubility, metabolic stability, and membrane permeability—using machine learning (ML) techniques. In this study, within the context of H2L optimization, we examined the applicability, accuracy, and utility of NES, a relatively new high-precision binding free energy calculation method, and evaluated its effectiveness in large-scale exploration of substituent space. The phosphodiesterase 9A inhibitor was used as a model system. Starting from the reported high-throughput screening hit compound, we first modified the core structure and then sequentially conducted large-scale exploration of two substitution sites. Following this protocol, we narrowed down compounds predicted to those exhibiting not only high binding affinity but also favorable physicochemical and ADME-related properties. Among these, we verified whether the lead compound reported in the literature was included, and confirmed that it appeared as one of the top-ranked candidates. These results demonstrate that an in silico protocol combining large-scale molecular generation, high-accuracy affinity prediction using NES, and ML-based ADME prediction enables H2L optimization that considers a broader substituent space.

**Graphical abstract:**

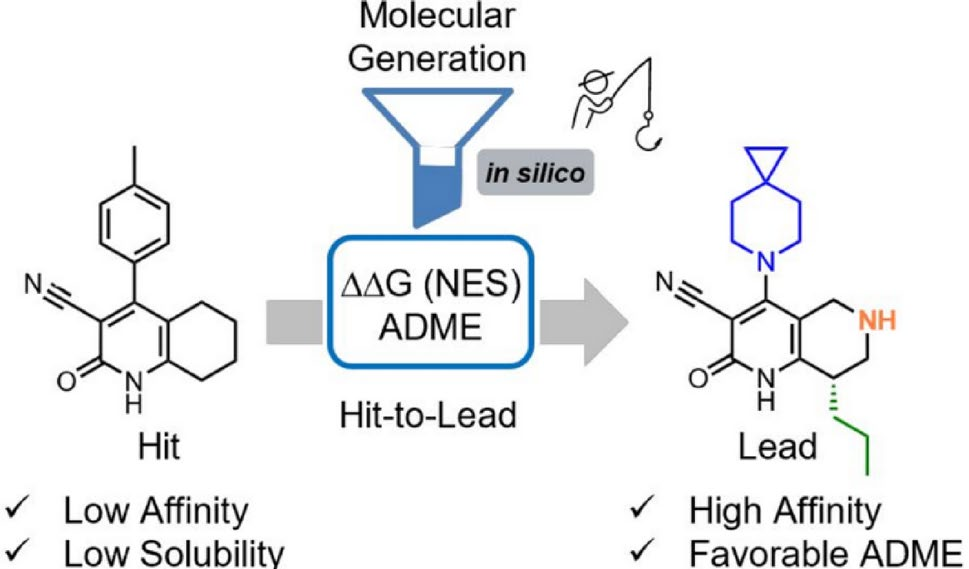

**Supplementary Information:**

The online version contains supplementary material available at 10.1007/s10822-025-00729-7.

## Introduction

Hit-to-lead (H2L) is a critical early stage in small-molecule drug discovery, where initial hit compounds are optimized into promising lead compounds. In this phase, hit compounds—identified through screening methods such as high-throughput screening (HTS) or virtual screening for their confirmed activity against a target protein—undergo optimization to enhance potency, selectivity, and absorption, distribution, metabolism, excretion, and toxicity (ADMET) properties [1–3]. Lead compounds are subjected to in vivo evaluation and serve as key decision points for advancing drug discovery projects. Further optimization may enable their progression into clinical candidates. Thus, identifying high-quality leads in the H2L stage significantly enhances the overall efficiency and success rate of drug discovery [4].

Conventionally, H2L has relied on experiment-driven workflows involving iterative cycles of synthesis and evaluation. However, due to practical limitations in compound synthesis, the chemical space explored is often restricted, leading to challenges in multi-objective optimization [5]. To address these limitations, in silico-driven H2L approaches have gained growing attention. These methods employ computational tools to generate large virtual libraries and prioritize compounds based on predicted binding affinity and ADMET properties, thereby enabling more efficient exploration of chemical space [6, 7]. An effective in-silico H2L process depends on three key components: (1) generation of diverse and chemically valid virtual compound series, (2) accurate prediction of binding affinity, and (3) reliable estimation of ADMET properties. Together, these elements facilitate the selection of compounds with balanced activity and ADMET properties, thereby increasing the likelihood of identifying promising leads.

Recently, molecular dynamics (MD)-based binding free energy calculations have become an increasingly efficient tool for predicting ligand–protein affinity, offering atomistic insights into binding mechanisms. Particularly, relative binding free energy (RBFE) calculations, such as Free Energy Perturbation (FEP) and Thermodynamic Integration (TI), are widely used in the H2L context [8–13]. RBFE calculations based on full-atom MD simulations incorporate solvent effects and molecular flexibility. These methods allow accurate prediction of binding affinity among structurally related compounds, typically within 1 kcal/mol of experimental values [14–18]. Hence, they are increasingly adopted in structure-based drug design to support compound selection [19–30]. Recent advancements in high-performance computing and cloud-based resources have made large-scale RBFE calculations feasible, allowing for high-accuracy screening across a broader chemical space [31–34]. Among these techniques, Non-equilibrium Switching (NES) has emerged as a promising RBFE method. A benchmark study by Gapsys et al. (2020) demonstrated that NES achieves predictive accuracy comparable to that of other RBFE approaches [35]. NES enables parallel execution of short MD simulations, offering excellent scalability for large-scale screening [36, 37]. However, the application of NES to H2L remains underexplored and warrants further investigation.

In H2L, optimizing physicochemical and ADMET properties is as critical as improving binding affinity. Accurate prediction of key properties—lipophilicity, aqueous solubility, metabolic stability, and membrane permeability—is particularly vital in early development stages. Leveraging large experimental datasets, machine learning (ML) models based on extended-connectivity fingerprints, and graph-based molecular descriptors have shown robust performance in this domain [38–42].

Despite improvements in predictive methods, the quality and diversity of the virtual compound libraries remain fundamental to the success of in silico-driven H2L. Even the best predictive models cannot identify suitable candidates if they are absent from the search space. Therefore, effective H2L approaches must systematically generate diverse synthetically accessible molecules that include comprehensive viable candidates. Molecular generation strategies encompass rule-based methods—R-group enumeration [43–46], reaction-based design [47–49], and matched molecular pair transformations [50–54]—as well as deep generative models [55–58]. While rule-based methods are classical, they offer the advantage of structural validity and synthetic accessibility by relying on well-established chemical transformation rules. They are especially effective for introducing a wide range of substituents to initial hit compounds. Moreover, RBFE methods produce optimal results when applied to congeneric series that share a core scaffold with variable substituents, making rule-based approaches, such as R-group enumeration, highly compatible with RBFE calculations.

**Fig. 1:**
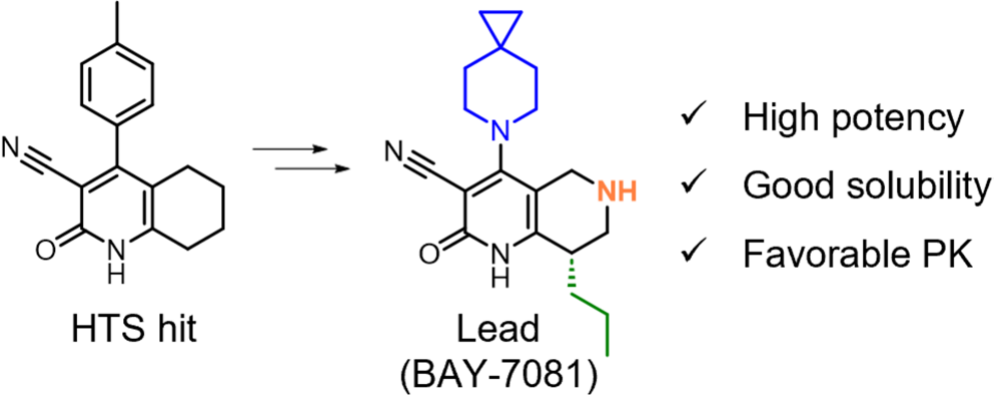
Chemical structures of the HTS hit and the optimized lead compound (BAY-7081)

In this study, we proposed an in silico-driven H2L protocol that integrates rule-based systematic molecular generation, high-accuracy RBFE calculations via NES, and ML-based property prediction. As a case study, we applied this workflow to an H2L campaign by Bayer, which identified BAY-7081 as a lead phosphodiesterase 9A (PDE9A) inhibitor with strong activity, good aqueous solubility, and a favorable pharmacokinetics profile (Fig. 1) [59]. We examined whether the proposed in-silico workflow could successfully identify the lead compound starting from the original hit. PDE9A, a cyclic guanosine monophosphate-specific phosphodiesterase, is considered a promising drug target for cardiovascular and central nervous system diseases [60]. We considered this case suitable for evaluating our in-silico protocol, as the original H2L process involved enhancing inhibitory activity, improving physicochemical properties, and scaffold hopping. The favorable pharmacokinetics profile of the lead compound was also attributed to reduced glucuronidation liability achieved through structural modifications. Our approach expanded chemical space via R-group enumeration, applied NES-based RBFE for affinity assessment, and employed ML models to predict key properties, such as lipophilicity and solubility. By integrating these components, the workflow enabled the selection of compounds with a favorable balance between potency and physicochemical and ADME properties. This study illustrates a practical implication of an in silico-driven H2L protocol and highlights its potential in enhancing lead discovery in future drug development.

## Methods

### Molecular generation

Molecular structures were generated through R-group enumeration based on a core scaffold. R-groups were selected from a substituent database analyzed by Bajorath et al. [45], who investigated common patterns of substituent transformations in bioactive small molecules. All substituents identified in these transformation patterns were initially included. From approximately 50,000 substituents in the database, we retained only those containing the elements C, H, O, N, P, S, or F. Substituents deemed unsuitable, such as those exhibiting chemical instability or susceptibility to rapid metabolism, were manually excluded. As a result, 30,477 R-groups were selected for further use (Table S1). The selected substituents were computationally introduced at specific positions of the core scaffold to generate a diverse set of derivatives.

### Protein preparation

The crystal structure of a PDE9A–inhibitor complex (PDB ID: 3JSW) [61] was used as the starting point for protein preparation, docking, and NES calculations. Protein preparation was conducted using the Protein Preparation Wizard [62], which added hydrogen atoms, assigned standard protonation states, and carried out a brief energy minimization. Four crystallographic water molecules coordinating Zn and Mg ions were retained.

We selected 3JSW as the receptor because it served as the structural basis for the docking model in the original Bayer H2L study, upon which our workflow is based. To evaluate structural consistency among available human PDE9A crystal structures, we superimposed 25 PDE9A entries, revealing high conservation across all chains (Fig. S1a). Although the overall backbone structures were highly conserved, moderate local structural differences were observed around the active site (Fig. S1b and c). To account for this local variation, NES calculations were also conducted using the 4Y86 structure [63], which differs from 3JSW in the active-site conformation. It was prepared the same way as 3JSW, retaining the crystallographic water molecules coordinating the Zn and Mg ions.

### Ligand preparation and docking calculations

All compounds were prepared using LigPrep [64], and Epik [65] (pH 7.4 ± 2.0) was employed to generate possible protonation states, tautomeric forms, and stereoisomers. Only neutral forms and tautomers specified as relevant to this study were kept for further processing. Docking simulations were conducted using Glide from Schrödinger [66–68]. The binding mode of the original HTS hit was determined using default docking with a van der Waals scaling factor of 0.6. A single docking pose was generated and adopted as the reference for subsequent docking studies. For all other compounds, core-constrained docking was employed to preserve the binding mode of the original HTS hit. A root-mean-square deviation threshold of 0.8 Å was used for core alignment, and a van der Waals scaling factor of 0.6 was applied. A single docking pose was generated for each ligand and used to construct the corresponding protein–ligand complex model. The constrained docking procedure consistently preserved the key hydrogen-bond interaction with Gln453. For all docking poses used as inputs for the NES calculations, we conducted visual inspections to confirm that this interaction was maintained and that the substituents at the hydrophobic pocket (H-pocket) were accurately positioned within the pocket. In contrast, unconstrained docking failed to retain the key hydrogen-bond interaction with Gln453 in several cases (Fig. S2 and S3; see Section S2 in the Supporting Information).

### Strain energy calculations

Local and global strain energy calculations were performed using MacroModel [69]. Local strain energy was determined by comparing tightly restrained and unrestrained minimizations of the docking pose; the energy difference between the two was taken as the local strain energy. All calculations were conducted using the OPLS_2005 force field [70]. Global strain energy was calculated by comparing the energy of the restrained docking pose with that of the lowest-energy conformer, which was obtained through a conformational search using the MCMM/LowMode method with the same force field.

### NES

RBFE calculations quantify the difference in binding affinity between two structurally similar ligands for a target protein. The RBFE (∆∆G_binding_ $\:[\text{A}\to\:\text{B}]$) between two ligands, A and B, is calculated using a thermodynamic cycle (Fig. S4). In this cycle, ∆G_complex_ (A→B) represents the free energy change for transforming ligand A into ligand B in the protein-bound state, and ∆G_solvent_ (A→B) is the corresponding transformation in solution. The RBFE is calculated as:

1 $$\begin{array}{*{20}c} {\Delta \Delta {\text{G}}_{{{\text{binding}}}} \left( {{\text{A}} \to {\text{B}}} \right) = ~\Delta {\text{G}}_{{{\text{complex}}}} \left( {{\text{A}} \to {\text{B}}} \right) - ~\Delta {\text{G}}_{{{\text{solvent}}}} \left( {{\text{A}} \to {\text{B}}} \right)} \\ \end{array} $$

In this study, RBFE values were computed using the NES method, which estimates free energy differences from multiple non-equilibrium transitions.

Ligands were parametrized with GAFF2 [71] (version 2.11) using ACPYPE [72] and AnteChamber [73] with AM1-BCC charges [74]. Simulation boxes were dodecahedral, with at least 1.5 nm between any atom and the box wall. TIP3P [75] water and Na⁺/Cl⁻ ions were added to achieve a 150 mM salt concentration. Protein parameters were assigned using the Amber99SB*-ILDN [76–78] force field, and ion parameters were taken from the work of Joung and Cheatham [79]. Each system was equilibrated using the following procedure. First, energy minimization was performed, followed by a 10 ps equilibration in the NVT ensemble at 298 K. Next, a 6 ns production run was conducted in the NPT ensemble at 298 K and 1 bar. After discarding the initial 2 ns, 80 snapshots were extracted at 47 ps intervals. Hybrid topologies were generated using pmx [80], and forward and backward alchemical transitions were performed starting from the 160 snapshots (80 snapshots for one ligand). Each non-equilibrium transition lasted 50 ps. A total of 20 ns of simulation time was used to compute each ∆∆G value in both bound and solvated states. Unless otherwise stated, three runs were conducted per ∆∆G calculation. Temperature was controlled using a velocity-rescaling thermostat [81] (τ = 0.1 ps), and pressure was maintained at 1 bar using the Parrinello–Rahman barostat [82] (τ = 5 ps). All bond lengths involving hydrogens were constrained using the LINCS algorithm [83]. Long-range electrostatic interactions were treated with the Particle Mesh Ewald method [84, 85], with a real-space cutoff of 1.1 nm, a Fourier grid spacing of 0.12 nm, and a relative interaction strength at the cutoff of 10^− 5^. Van der Waals interactions were smoothly switched off between 1.0 and 1.1 nm, and dispersion corrections were applied to both energy and pressure. During alchemical transitions, non-bonded interactions were handled using a modified soft-core potential [86]. All simulations were performed with GROMACS 2024.1 [87]. For each transition, work values were calculated from Hamiltonian derivatives along λ. The Crooks’ Fluctuation Theorem [88] was applied with a maximum likelihood estimator to convert non-equilibrium work distributions into equilibrium free energy differences. The ∆∆G values were calculated as the mean and standard deviation across runs. Two types of perturbation map strategies were employed: a star map, in which all compounds are connected directly to a common reference compound, and a cycle-closure map, in which compounds are connected in multiple closed loops to enable internal consistency checks through thermodynamic cycles. While the star map allows broad compound coverage with minimal connections, the cycle-closure strategy offers enhanced accuracy by leveraging redundant pathways for error correction. Only runs with more than ten valid transition paths were included in ∆∆G calculations. For cycle-closure calculation, a perturbation map was generated using FEP+ [89] with a similarity score cutoff of 0.3, based on both 2D and 3D molecular similarities. In cases of failed ∆∆G calculations, transitions were repeated with a reduced time step of 0.5 fs to improve convergence. Final ∆G values for each compound were obtained using Cinnabar (https://github.com/OpenFreeEnergy/cinnabar), based on the computed ∆∆G values. To evaluate the efficiency of substituent modifications in improving binding affinity, we introduced the Substituent Binding Efficiency (SBE) index, defined as:

2 $$ SBE = \Delta \Delta {\text{G/}}\Delta {\text{HA}} $$

where ΔΔG represents the RBFE from the reference compound, and ΔHA is the difference in heavy atom count. To emphasize the exceptional efficiency of structure-invariant gains in cases where ΔHA = 0 and ΔΔG < 0, the SBE was assigned using the following adjusted definition:

3 $$ {SBE = \Delta \Delta G \times 2} $$

No cases with ΔHA < 0 were observed in this study; thus, SBE was evaluated only for modifications with ΔHA ≥ 0.

### Physicochemical and ADME properties prediction

**Fig. 2:**
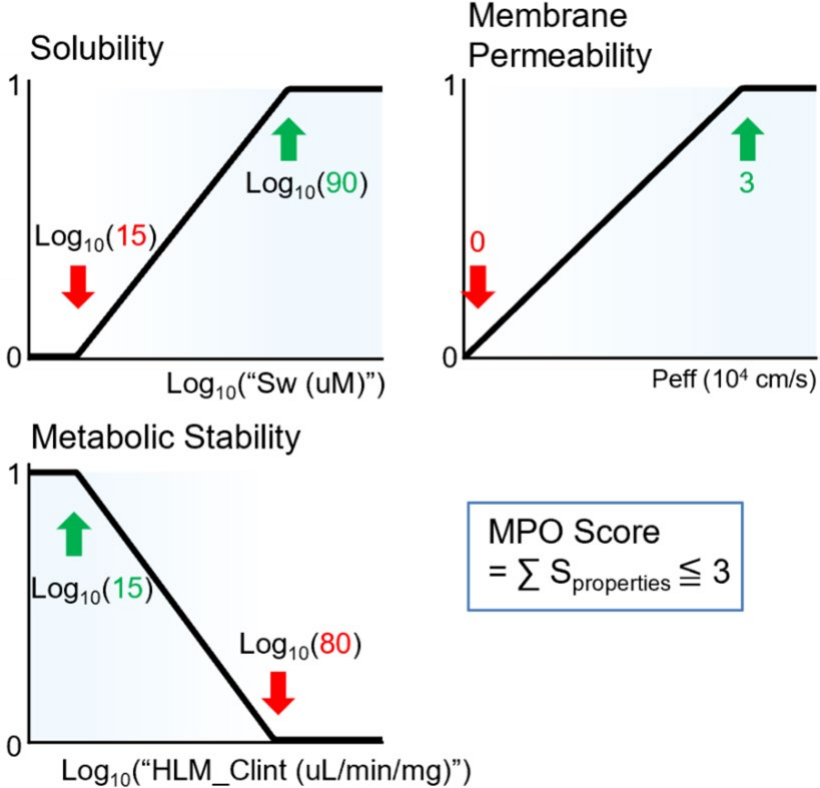
Scoring scheme used for MPO calculation. Up to one point each is assigned to solubility, metabolic stability, and membrane permeability, yielding a maximum total score of 3

Physicochemical and ADME properties predictions were performed using ADMET Predictor version 11.0 [90]. Predicted properties included octanol–water partition coefficient (LogP) as lipophilicity, aqueous solubility in pure water (Sw) as solubility, in vitro intrinsic clearance in human liver microsome (HLM_Clint) as metabolic stability, and effective jejunal permeability (Peff) as membrane permeability. As shown in the study [91], the predictive accuracy of these properties is considered sufficient for application in early-stage drug discovery. While ADMET Predictor employs regression-based models for these predictions, the referenced study assessed prediction performance using classification criteria. Based on threshold values defined in that study, we constructed a scoring function that assigns up to one point each for favorable predictions of solubility, metabolic stability, and membrane permeability (Fig. 2). The Multi-Parameter Optimization (MPO) score is defined as follows:

4 $$ \begin{aligned} MPO\;~Score & = S_{{solubility}} \\ & \quad + S_{{metabolic~stability}} + S_{{membrane~permeability}} \\ \end{aligned} $$

where *S*_*solubility*_, *S*_*metabolic stability*_, *and S*_*membrane permeability*_ represent the scaled scores for solubility, metabolic stability, and membrane permeability, respectively. The MPO score has a maximum value of 3 and serves to evaluate the overall balance among the three ADME properties.

## Results and discussion

### Overview

In this study, we proposed an in silico-driven H2L protocol by integrating molecular generation, binding affinity evaluation, and ADME property prediction, and applied it to PDE9A inhibitors reported by Bayer as a case study.

**Fig. 3:**
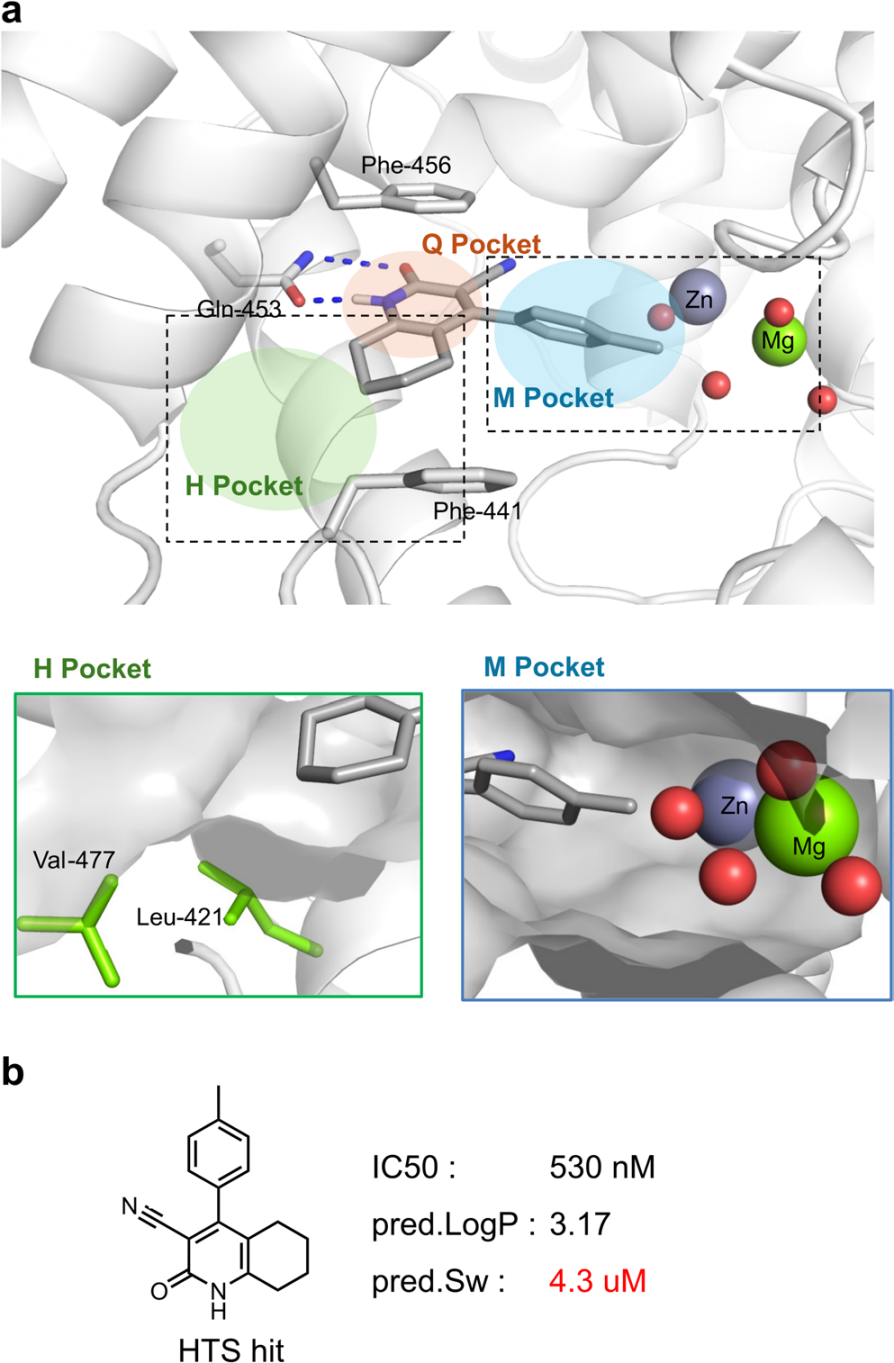
PDE9A binding site and the HTS hit compound. **a** Docking pose in the crystal structure of human PDE9A (PDB ID: 3JSW). The H pocket, a relatively small pocket enclosed by Val477 and Leu421, is unoccupied. The M pocket accommodates the p-tolyl substituent, which is located near a water molecule coordinated to Zn and Mg ions. **b** The IC_50_ value is taken from the literature, while LogP and Sw represent predicted physicochemical properties

The PDE9A binding site comprises three sub-pockets: the Q pocket, which contains Gln453 and facilitates hydrogen-bonding interactions; the H pocket, composed of lipophilic residues; and the M pocket, which extends into the solvent-exposed region. Docking results indicated that the core structure of Bayer’s hit compound formed a hydrogen bond with Gln453 in the Q pocket, while a p-tolyl substituent occupied the M pocket. The hydrophobic H pocket remained unoccupied, suggesting that a substituent in this pocket could enhance binding interactions (Fig. 3a). This hit compound exhibited moderate inhibitory activity (IC₅₀ = 530 nM), while property predictions indicate high lipophilicity and poor aqueous solubility (Fig. 3b). Given the hydrophobic nature of the H pocket, introducing hydrophobic substituents was expected to enhance binding affinity. However, the incorporation of hydrophilic substituents to improve physical properties, such as aqueous solubility, is likely to be challenging. Conversely, the M pocket, which faces the solvent region, is expected to accommodate diverse substituents. This suggests that replacing the p-tolyl group could potentially adjust the physicochemical properties. In the Q pocket, a portion of the core of Bayer’s hit compound is exposed to the solvent region, suggesting the potential for introducing hydrophilic modifications to the core to improve physicochemical and ADME properties. Therefore, the docking model of Bayer’s hit compound suggests a strategy for molecular design: selecting a core structure with favorable ADME properties, introducing hydrophobic substituents into the H pocket to enhance activity (despite potential ADME trade-offs), and identifying M-pocket substituents that achieve a balanced compromise between activity and ADME properties.

**Fig. 4:**
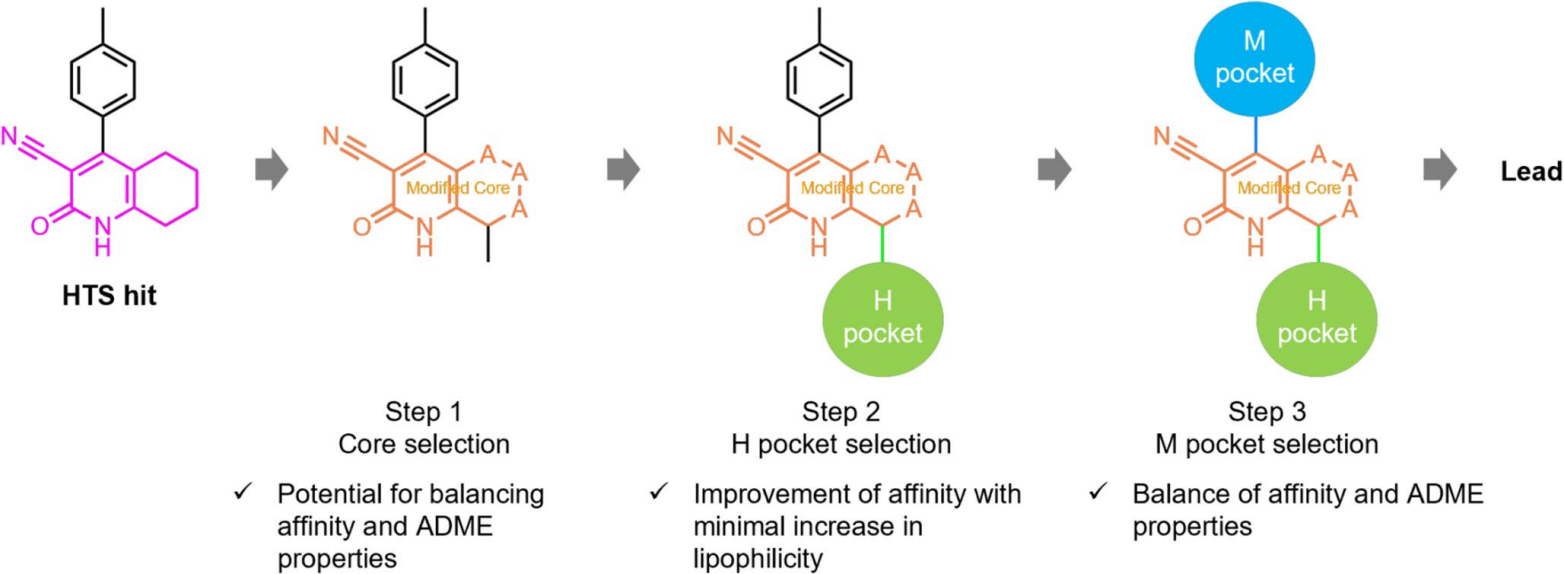
Overview of the proposed in-silico H2L protocol

Based on these considerations, we designed a three-step in silico-driven H2L protocol to identify lead compounds that exhibit both high binding affinity and favorable physicochemical and ADME profiles (Fig. 4):

- *Step 1*. Core selection based on predicted activity and predicted physicochemical and ADME profiles.
- *Step 2*. H-pocket substituent selection to enhance binding affinity with minimal increase in lipophilicity.
- *Step 3*. M-pocket selection to retain or improve predicted binding affinity, while improving predicted physicochemical and ADME properties

Subprotocols for Steps 2 and 3 have been proposed and are detailed in their respective sections.

### Assessment of NES performance prior to compound exploration

**Fig. 5:**
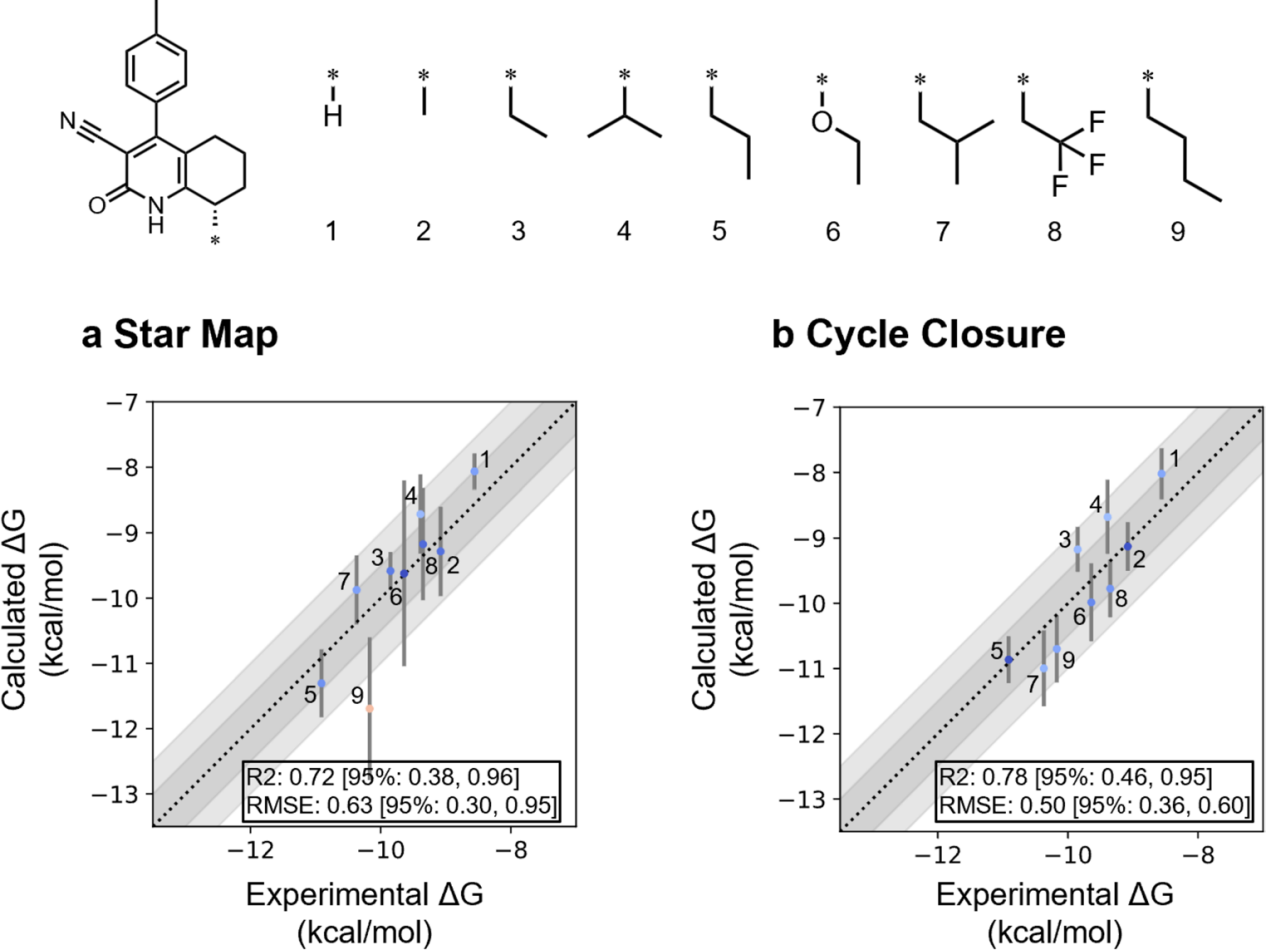
NES calculations for the H pocket using literature compounds. Compound 1 was used as the reference in the star map. The cycle-closure map was generated using FEP+, and accuracy was evaluated using R² and RMSE. The gray bands represent ± 0.5 and ± 1 kcal/mol from the ideal. Vertical error bars indicate the standard deviations of NES results, while the 95% confidence intervals for R² and RMSE were estimated by bootstrap resampling (*n* = 1000)

Before compound exploration in Steps 1–3, we assessed the NES performance for PDE9A inhibitors reported in Bayer’s study for both the H and M pockets. First, targeting the H pocket, NES was applied to nine derivatives of the HTS hit. Two perturbation strategies—star map and cycle closure—were compared. NES showed good correlation with both strategies (Fig. 5), while the cycle closure exhibited superior predictive performance (R² = 0.78, root mean square error [RMSE] = 0.50) relative to the star map (R² = 0.72, RMSE = 0.63). The cycle closure also yielded lower standard deviations, indicating reduced variability across runs and enhanced reproducibility. Additional comparisons with FEP+ and OpenFreeEnergy (OpenFE) [92] further confirmed the comparable accuracy of NES (Fig. S5). These results suggest that NES, particularly when using cycle closure, provides reliable and stable predictions of binding affinity for the H pocket.

To assess the sensitivity of NES results to receptor conformation and docking pose selection, we conducted additional validation studies. We performed NES validation using the 4Y86 structure, which features a shorter Ala452–Leu420 distance and an alternative Tyr424 conformation predicted to influence the H-pocket environment (Fig. S1c). Compared with 3JSW, the predictive accuracy was slightly lower in both the star map and cycle closure, although the latter again exhibited smaller standard deviations of predicted affinities across compounds (Fig. S6a and b). Despite these local conformational differences, their impact on the overall predictive performance was minor. Given the slightly higher correlation obtained with 3JSW, we concluded that this receptor is suitable for use in the in-silico H2L protocol.

To further examine pose sensitivity, NES calculations were performed using five docking poses for 3JSW as initial structures for each of compounds 4–9 to evaluate pose sensitivity (Fig. S7). Although some poses with lower docking scores yielded larger prediction errors, most calculated affinities were within ± 1 kcal/mol of the experimental values. These findings suggest that, while docking pose variations can influence predicted values to some extent, they do not substantially affect the overall ranking of potent compounds. Collectively, our results demonstrate that although receptor state and pose selection can influence absolute accuracy, NES remains reasonably robust in ranking compounds and identifying high-affinity candidates.

**Fig. 6:**
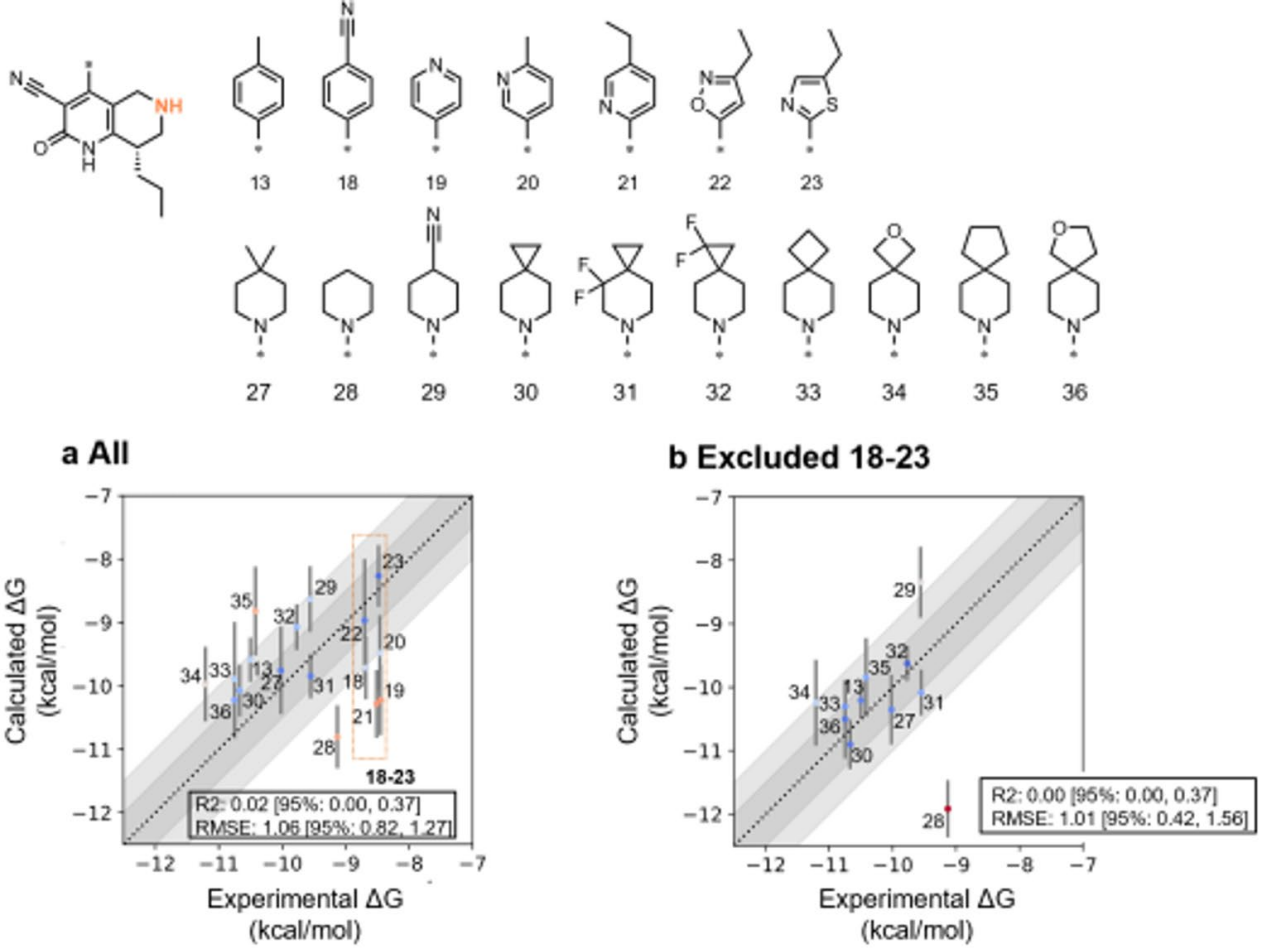
NES calculations for the M pocket using compounds reported in the literature. **a** NES prediction results for all compounds. **b** Results excluding compounds 18–23. In both panels, the cycle-closure perturbation map was generated using FEP+. The gray bands represent ± 0.5 and ± 1 kcal/mol from the ideal. Vertical error bars indicate the standard deviations of NES results, while the 95% confidence intervals for R² and RMSE were estimated by bootstrap resampling (*n* = 1000)

Next, targeting the M pocket, NES was applied to 17 derivatives featuring a core distinct from the HTS hit. A perturbation map employing the cycle-closure approach was generated, followed by NES calculations. Overall, the predictive accuracy of M-pocket transformations was low, particularly for compounds with aromatic substituents (18–23) and compound 28 (Fig. 6a). Quantum mechanics-based tautomer predictions were performed using Jaguar [93, 94], indicating that, for compounds 18–23, the dominant tautomer differed from the bioactive tautomer (Fig. S8). This alternative tautomer lacked interactions with Gln453, possibly leading to reduced binding affinity. Thus, NES likely overestimated the affinities of these aromatic compounds due to the low population of the bioactive tautomer. The tautomeric issue associated with aromatic substituents is likely attributable to the structural conversion of the core scaffold from the HTS hit. In contrast, compounds with aliphatic substituents were predicted to predominantly exist in the bioactive tautomeric form, resulting in relatively accurate and reasonably consistent predictive performance for affinities. Upon exclusion of compounds 18–23, cycle-closure NES calculations indicated that compound 28 remained overestimated, while the predicted binding free energies of the remaining compounds were within ± 1 kcal/mol of the experimental values (Fig. 6b). Calculations performed using FEP+ and OpenFE exhibited a similar trend, with compounds bearing aliphatic substituents showing good correlation with experimental values, while those with aromatic substituents tended to overestimate binding free energy (Fig. S9). Notably, compound 28 was not overestimated by either FEP+ or OpenFE, suggesting that the observed deviation may be specific to the NES method. While the binding affinity of a certain compound was overestimated, potentially leading to false-positive compounds, these results were considered to support the applicability of NES to identify promising M-pocket substituents in the exploration of aliphatic derivatives when the core scaffold is modified.

Summarizing these results for both H and M pockets, NES offers sufficient predictive accuracy to be used in compound selection during Steps 1–3.

### Core selection (Step 1)

**Fig. 7:**
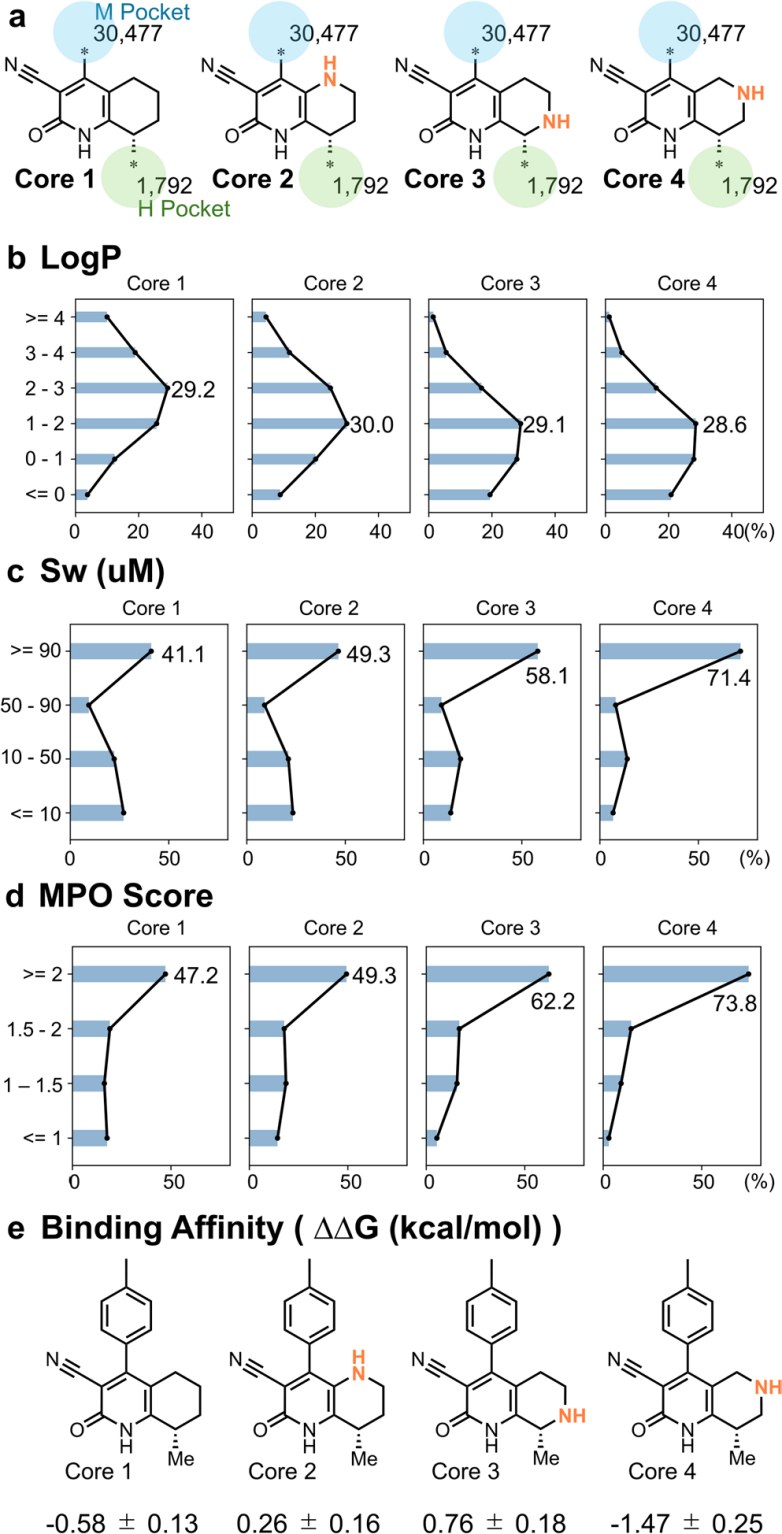
Predicted property distributions and binding affinities for Cores 1–4. **a** Combinatorial compound generation using 1,792 H-pocket and 30,477 M-pocket substituents. **b**–**d** Distributions of predicted LogP, solubility (Sw), and MPO score. **e** Binding affinities (ΔΔG) predicted by NES; the Core 1 with hydrogen for the H pocket was used as the reference (ΔΔG = 0)

Compound exploration for PDE9A inhibitors was initiated from core selection (Step 1) (Fig. 7). We selected four core structures (Cores 1–4) based on structure–activity relationship data reported in the literature. The objective of step 1 was to identify core structures that exhibited both favorable predicted binding affinity and desirable ADME properties.

To assess the ADME properties of the core structures, we introduced a series of substituents into each core, predicted their physicochemical and ADME characteristics, and compared their distribution profiles. Given that the H pocket is a small lipophilic site, we initially selected hydrophobic substituents suitable for this region. Starting from the HTS hit compound (Core 1), we generated molecules and filtered them based on docking scores and strain energy, yielding 1792 candidate substituents for the H pocket (Fig. S10). These substituents were combinatorially paired with 30,477 M-pocket substituents for each core, generating approximately 216 million compounds. We then predicted physicochemical and ADME properties for the enumerated compounds and compared their distributions across the four cores (Fig. 7a–d). Cores 2–4 showed lower lipophilicity (LogP) than Core 1, with Core 4 demonstrating notably improved aqueous solubility. Additionally, Core 4 also showed favorable MPO score distributions, with over 70% of compounds scoring ≥ 2, indicating that most compounds exhibited favorable solubility, metabolic stability, and membrane permeability profiles.

To assess binding affinity, NES calculations were performed on representative compounds from each core (Fig. 7e and Fig. S11). Specifically, the full cycle-closure NES was applied to compounds with hydrogen and methyl substituents for the H pocket and p-tolyl group for the M pocket, respectively. Among the Cores 1–4, Core 4 was predicted to have the highest binding affinity (Fig. 7e).

Based on these findings, Core 4 was selected for further investigation due to its optimal balance of predicted binding affinity and favorable physicochemical and ADME properties.

### H-pocket substituent exploration (Step 2)

**Fig. 8:**
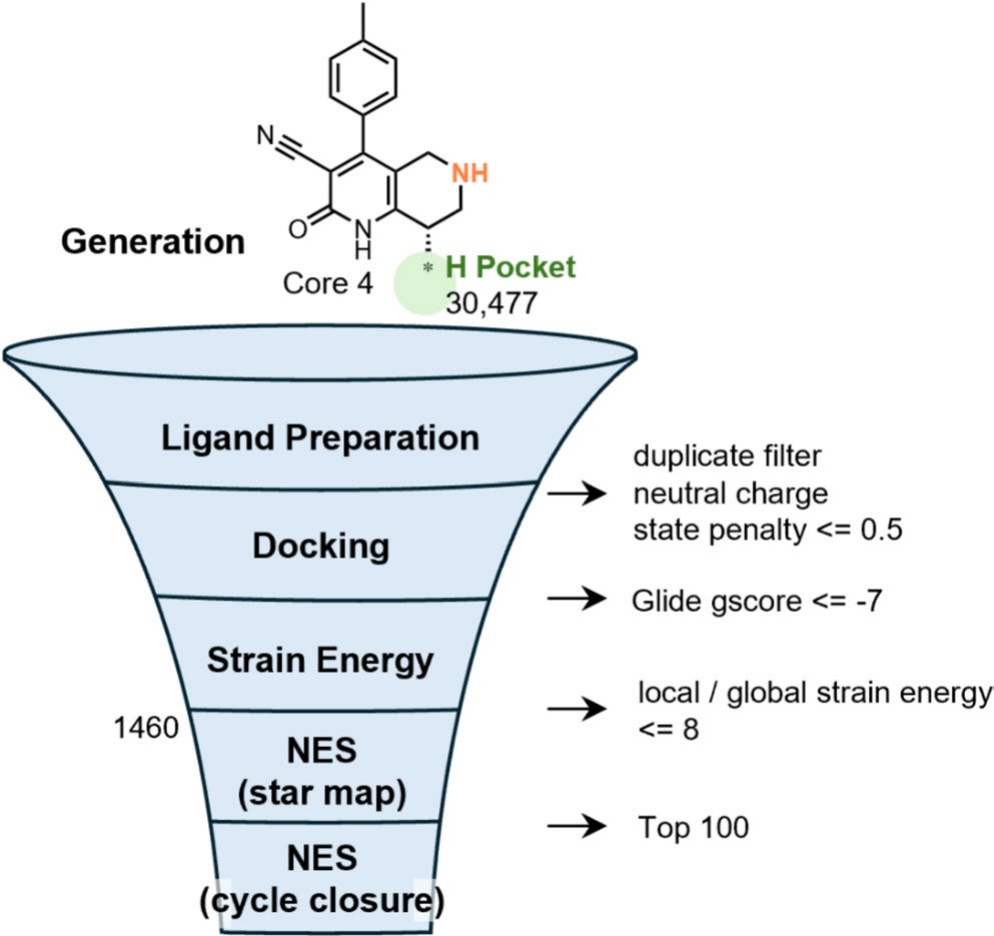
Workflow for H-pocket exploration in Core 4. Substituents were generated via R-group enumeration and filtered based on their ligand properties, docking scores, and strain energy. NES calculations were first performed on 1460 compounds using the star map approach (one run per edge), followed by cycle-closure NES calculations (three runs per edge) for the top 100 compounds

In Step 2, we explored the H pocket of Core 4 to improve binding affinity by identifying optimal substituents (Fig. 8). Given the poor solubility of the HTS hit, we determined that avoiding the introduction of excessively hydrophobic substituents into the H pocket was crucial to prevent obtaining highly active yet poorly soluble compounds. Therefore, physicochemical properties such as solubility and MPO score were also evaluated. Additionally, the binding free energy change per heavy atom (SBE) was considered as a complementary metric to prioritize efficient substituents. Using the workflow outlined in Fig. 8, we initially generated 30,477 compounds and subsequently selected candidates through sequential filtering based on ligand properties, docking scores, and strain energy. We then conducted a rapid NES screening (one run per edge) on 1,460 compounds using a star map, with the unsubstituted compound as the reference. The top 100 candidates were further evaluated using NES with cycle closure (three runs per edge), also using the unsubstituted compound as the reference.

**Fig. 9:**
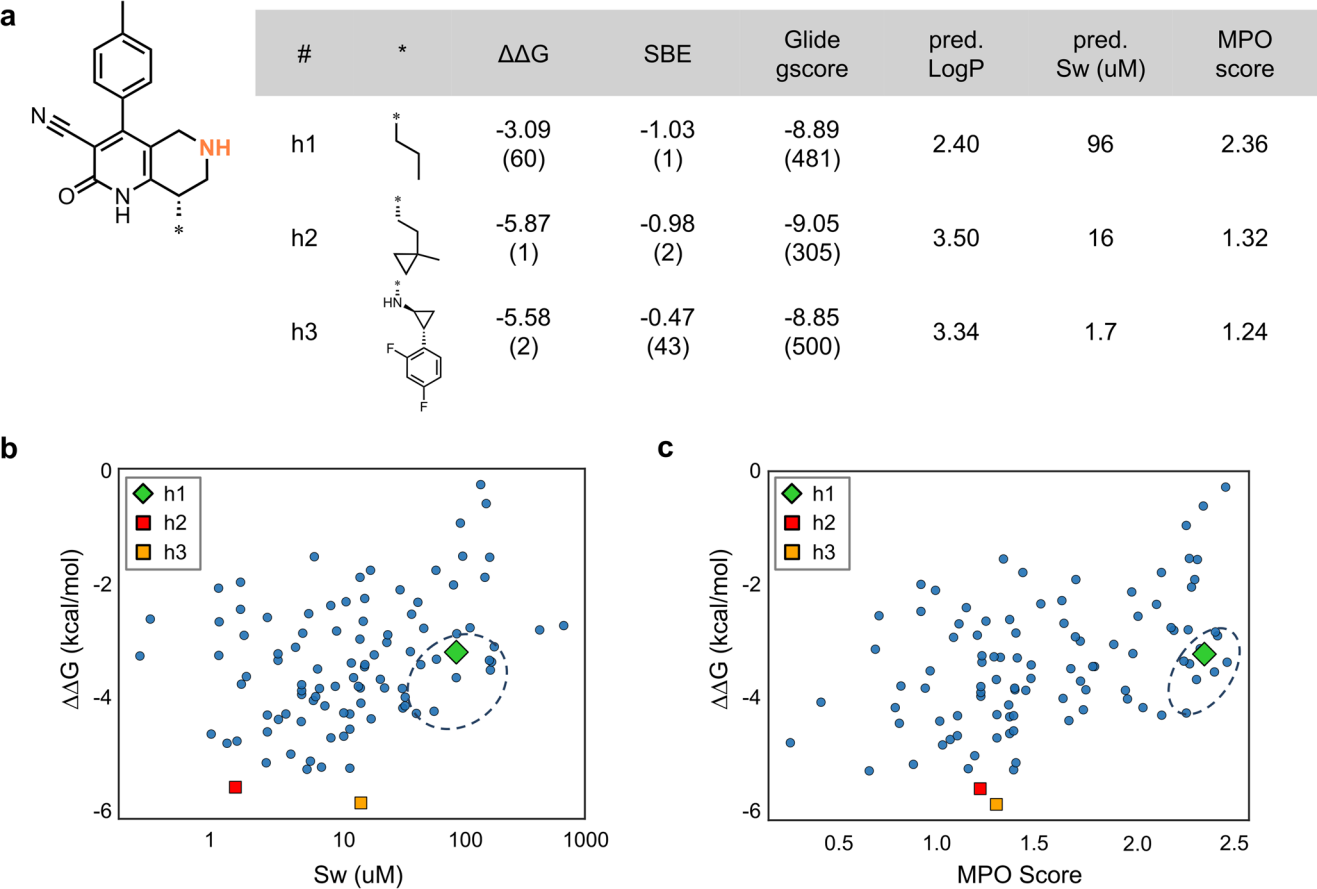
Summary of H-pocket substituent exploration for Core 4. **a** Representative substituents with high predicted binding affinity. ΔΔG values (kcal/mol) were calculated using NES with cycle closure, with the unsubstituted compound used as the reference (ΔΔG = 0). Values in parentheses indicate rankings among the 1460 compounds. **b** Scatter plot of predicted solubility versus binding affinity (ΔΔG). **c** Scatter plot of MPO score versus binding affinity (ΔΔG). In both **b** and **c**, the dashed circles highlight the region where favorable binding affinity is achieved in conjunction with either high solubility or high MPO score

As shown in Fig. 9a, the n-propyl (nPr) group (h1), which is present in the lead compound, ranked 60th in the NES binding free energy evaluation and was a well-balanced substituent offering a desirable combination of predicted binding affinity and physicochemical properties. While several compounds exhibited strong predicted binding affinities based on NES with cycle closure, many of these high-ranking candidates, such as h2 and h3, had unfavorable properties, including low predicted solubility and low MPO score. This trend illustrates a common trade-off: enhanced binding through hydrophobic interactions often comes at the cost of reduced aqueous solubility. Notably, the nPr group (h1) was one of the few substituents that successfully achieved strong predicted binding affinity with both high aqueous solubility and a high MPO score. It was located in the lower-right region of the binding affinity vs. solubility plot (Fig. 9b), indicating a favorable balance of low ΔΔG and relatively high aqueous solubility. In the ΔΔG versus MPO score plot (Fig. 9c), it also fell into the desirable low-ΔΔG, high-MPO region, suggesting good overall ADME properties. Moreover, from the perspective of SBE, the nPr group ranked highest among the 100 compounds evaluated with NES with cycle closure. The addition of only three heavy atoms resulted in a substantial affinity gain without significantly increasing lipophilicity. Taken together, these findings demonstrate that the nPr group serves as a rare and valuable H-pocket substituent, offering strong predicted binding affinity, high solubility, and favorable ADME properties. While it showed low priority in docking-based ranking (470th), NES-based free energy prediction was able to recognize its favorable balance of affinity and ADME properties. These findings emphasize the added value of NES in early-stage affinity prediction and compound prioritization.

### M-pocket optimization (Step 3)

**Fig. 10:**
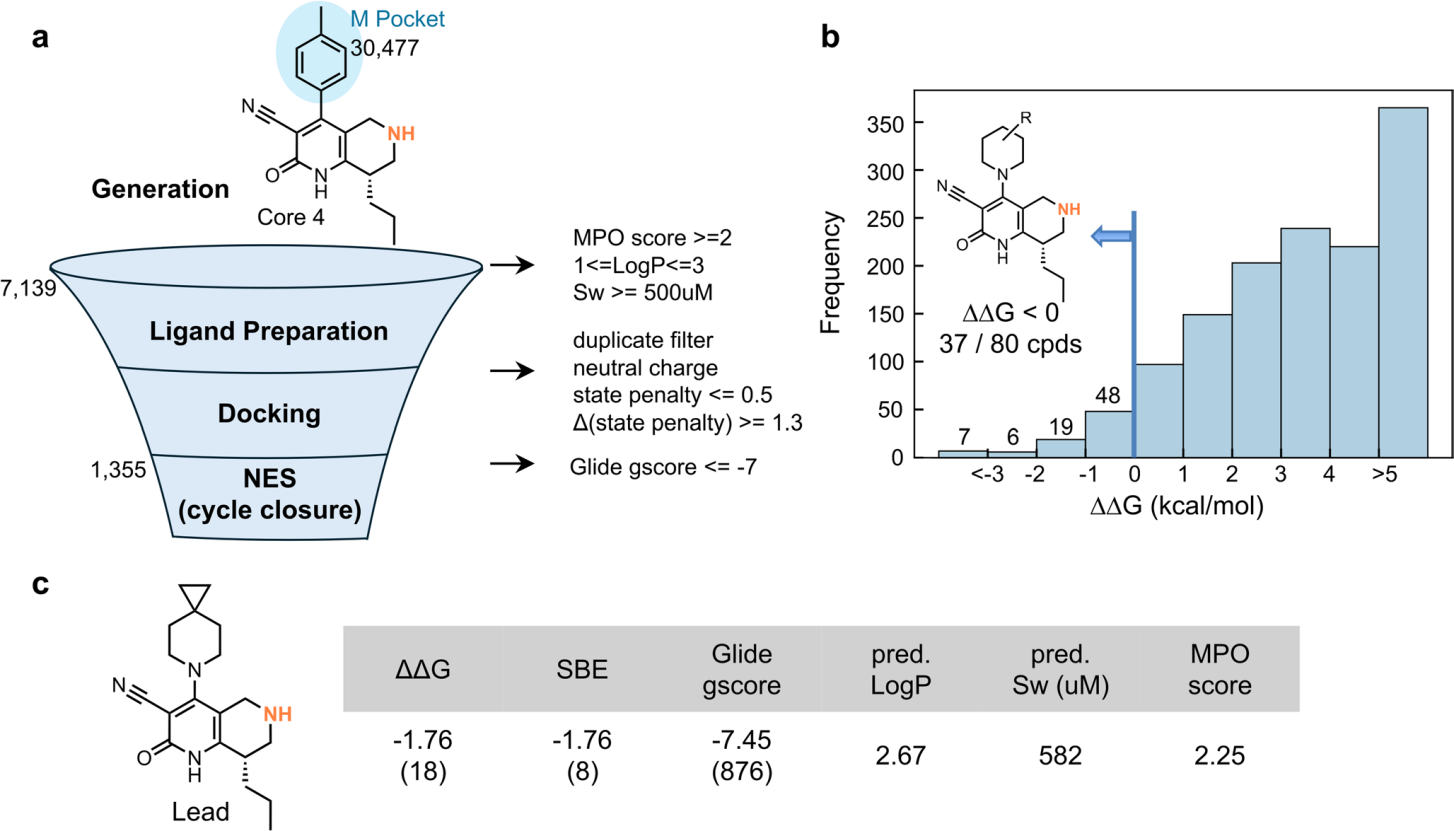
Results of M-pocket optimization of Core 4. **a** Workflow for molecular generation and evaluation. **b** Distribution of predicted ΔΔG values from NES calculations. The compound bearing a toluene group in the M pocket was used as the reference (ΔΔG = 0). A total of 80 compounds showed ΔΔG < 0, of which 37 contained a piperidine ring. **c** Predicted properties of the lead compound. Values in parentheses indicate rankings among the 1355 compounds

In Step 3, we focused on optimizing the M-pocket substituents while maintaining the nPr group in the H pocket of Core 4 (Fig. 10). Following the affinity gains from H-pocket optimization, this step aimed to identify M-pocket substituents with comparable or improved binding affinity and favorable physicochemical and ADME profiles. Using the workflow shown in Fig. 10a, we generated Core 4 derivatives with diverse M-pocket substituents. From this set, 7,139 compounds were selected based on favorable ADME properties (MPO score ≥ 2), suitable lipophilicity (1 ≤ LogP ≤ 3), and markedly improved solubility compared to the HTS hit (Sw ≥ 500 µM). To ensure consistency of tautomeric states during NES calculations, molecules were selected based on large differences in Epik state penalties between two possible tautomers, such that one tautomer was strongly favored. Subsequently, molecular docking, followed by NES cycle-closure calculations (one run per edge), was performed on a subset of 1,355 compounds exhibiting favorable docking scores. In these NES calculations, the M-pocket substituent of the reference compound was the p-tolyl group, which corresponds to the substituent of the HTS hit compound. Among the 1,355 compounds, only 80 compounds demonstrated a lower ΔΔG than the reference (Fig. 10b). Notably, 37 of these compounds contained the piperidine moiety, suggesting that piperidine derivatives represented a dominant chemotype among the high-affinity candidates. The lead compound reported by Bayer ranked 18th in NES-based affinity predictions but only 876th by docking score (Fig. 10c), again emphasizing the superior predictive accuracy of NES, as demonstrated in the H-pocket study. In addition to its high predicted affinity, the lead compound exhibited well-balanced physicochemical characteristics, including suitable lipophilicity, high aqueous solubility, and a favorable ADME profile (MPO score ≥ 2). These results underscore the compound as a promising lead, demonstrating both strong predicted binding activity and favorable physicochemical and ADME properties.

### Glucuronidation risk assessment

The in silico-driven H2L protocol successfully prioritized the lead compound (BAY-7081) among the top-ranked candidates, demonstrating its effectiveness in identifying compounds with both high predicted activity and favorable physicochemical and ADME properties. However, previous experimental studies have indicated that incorporation of a piperidine moiety into the M pocket of the lead compound was also intended to reduce the risk of glucuronidation, a metabolic liability not directly addressed in our selection criteria. To investigate this aspect, we attempted an in-silico evaluation of metabolism mediated by uridine 5′-diphospho-glucuronosyltransferase. Although existing prediction tools provided qualitative insights into glucuronidation potential, no clear threshold could be established to reliably differentiate compounds with high or low glucuronidation liability. A more detailed analysis, including glucuronidation risk predictions, is provided in Section S3 of the Supporting Information (Fig. S12). The development of an effective predictive model for glucuronidation risk would be beneficial for establishing a more robust protocol for in silico-driven H2L in future research.

### Advantages and limitations of in silico-driven H2L optimization

A key advantage of the in-silico H2L protocol lies in its capacity to efficiently screen a diverse array of substituent candidates from an expanded chemical space, thereby facilitating a comprehensive evaluation of compounds that balance target activity with physicochemical and ADME profiles. Accurate binding affinity predictions obtained through NES-based RBFE calculations enabled the identification of promising substituents that would have been difficult to prioritize using conventional docking methods. Notably, the lead compound was successfully ranked among the top candidates, underscoring the effectiveness of this approach. As discussed earlier, the H-pocket exploration revealed that only a limited number of substituents exhibited both high predicted affinity and favorable ADME profiles (Fig. S13). Among these, approximately 10 substituents, including the most promising nPr group, showed such a balance.

In the M-pocket exploration, initial filtering based on predicted ADME properties using ML ensured that all final candidates possessed acceptable ADME characteristics, among which 80 compounds showed high predicted binding affinities. These results demonstrate that only a small number of substituents met the dual criteria of predicted affinity and ADME properties in each pocket, emphasizing the importance of efficient computational prioritization. Compared to experiment-driven H2L workflows, this protocol not only expands the searchable chemical space but also enables efficient narrowing of candidates to a focused subset suitable for experimental validation. This capability also helps to accelerate the overall H2L process compared to conventional experiment-driven workflows. In our study, NES-based computational screening was conducted over 1 month using approximately 120,000 graphics processing unit (GPU) hours on scalable cloud infrastructure, enabling the rapid identification of high-quality candidates from a diverse chemical space.

The NES-based framework offers practical computational advantages over conventional FEP-based H2L campaigns. Within the context of previous large-scale RBFE-based H2L campaigns, such as those utilizing FEP+ for EGFR [27], DLK [28], and Wee1 inhibitors [29], our NES-driven protocol offers distinct computational and methodological advantages. FEP+ depends on replica-exchange simulations that require synchronous execution across λ-windows, thereby limiting scalability and responsiveness. In contrast, NES leverages independent short non-equilibrium switching simulations, enabling flexible parallelization and somewhat faster turnaround for free-energy estimation while maintaining comparable predictive accuracy. Notably, the NES method is implemented in open-source molecular simulation packages, facilitating transparent, reproducible, and extensible workflows that are readily adoptable across diverse research environments.

To our knowledge, large-scale applications of non-equilibrium free-energy calculations in H2L optimization remain rare, with the COVID Moonshot project [33] being the only previously reported example. In that study, long non-equilibrium simulations enabling the star-map analysis were performed using exascale computing resources that are normally unavailable. In contrast, our workflow implemented a practical two-step NES strategy that combines rapid star-map screening with precise cycle-closure calculations, without relying on excessive computational resources. This demonstrates that NES can serve as a scalable, open-source, and computationally accessible approach for realistic H2L optimization. Furthermore, our workflow integrates ML-based ADME property prediction directly with NES-derived affinity estimation. Whereas previous FEP-based studies have primarily employed physicochemical filters in their consideration of ADME properties, our integrated protocol is characterized by its ability to directly estimate multiple ADME properties—such as membrane permeability, metabolic stability, and aqueous solubility—in silico, and to optimize them simultaneously with potency using NES. Although ML models for property prediction have been adopted in H2L optimization [42] or introduced as platform frameworks [39], these studies have generally assessed ADME properties independently of large-scale free energy simulations. To our knowledge, this study is among the first to explicitly combine large-scale free energy calculations with ML-based property prediction within an integrated H2L workflow, providing a clear example of how in silico lead optimization can holistically balance activity and ADME properties.

Despite these advantages, the current workflow has practical and methodological limitations that warrant further validation. First, the current in-silico protocol was applied only to the PDE9A inhibitor series reported by Bayer, and experimental validation of the top-ranked compounds has not yet been performed. Therefore, the predictive performance of the activity and property models remains to be fully verified, and future studies are necessary to evaluate the generalizability of the workflow to other target systems. Second, the success of this protocol inherently depends on the predictive accuracy of the underlying models. Its applicability is therefore limited to target systems for which reliable free-energy calculations can be performed and to chemical series for which valid ADME property-prediction models are available. Moreover, for certain ADME-related properties that are critical for H2L optimization—such as glucuronidation liability—robust predictive models are still lacking, which limits the comprehensiveness of in-silico evaluation. Therefore, when implementing an in-silico protocol for H2L optimization, it is essential to validate and refine the models as experimental data become available. Overall, the finding that the existing lead compound was evaluated as promising through this protocol in the case of PDE9A inhibitor optimization suggests that an in silico–driven workflow integrating activity prediction by NES and ADME property prediction by ML represents a promising option for H2L optimization across vast chemical space.

## Conclusion

In this study, we proposed an in silico-driven H2L protocol using PDE9A inhibitors as a model system. This protocol integrates molecular generation, NES-based binding affinity prediction, and ML-based physicochemical and ADME property prediction. We began by identifying core scaffolds that exhibited a favorable balance between predicted binding affinity and ADME characteristics. Subsequently, in the H pocket, substituents were prioritized based on the simultaneous achievement of high predicted binding affinity and favorable ADME properties. Additionally, the binding free energy change per heavy atom (SBE) was considered to prioritize efficient substituents with minimal increase in lipophilicity. In the M pocket, substituents were prioritized using NES-derived affinity and ADME evaluation, successfully ranking the known lead compound among the top candidates. A key advantage of this protocol lies in its capacity to efficiently screen a diverse array of substituent candidates from an expanded chemical space, facilitating a comprehensive evaluation of compounds that balance target activity with physicochemical and ADME profiles. Furthermore, the computational workflow, which required approximately 120,000 GPU hours, was completed within 1 month using scalable cloud infrastructure, demonstrating the potential to accelerate lead identification relative to conventional, experimentally driven H2L processes.

Despite these advantages, the current workflow has practical and methodological limitations that warrant further validation. It was applied only to the PDE9A inhibitor series reported by Bayer, and experimental confirmation of the top-ranked compounds, as well as evaluation of the workflow’s applicability to other targets, remains necessary. Additionally, the validity of this protocol depends on the availability of accurate methods for predicting both activity and ADMET properties. While this study demonstrates that NES-based binding affinity prediction can serve as a reliable approach for evaluating large compound sets, its accuracy still needs to be verified across chemically and biologically diverse systems. Moreover, the ADMET properties required for lead generation differ depending on the specific project and the characteristics of each HTS hit compound, necessitating prediction models tailored to these variations. Certain endpoints—such as glucuronidation liability—currently lack robust and validated predictive models, representing a limitation of current in-silico approaches. Continued refinement of both affinity and ADMET prediction methodologies, guided by experimentally measured data, will be essential to enhance the reliability of computational H2L workflows. In this context, close integration between experimental and computational efforts will be critical to fully realize the potential of in silico-driven H2L optimization for accelerating the discovery of high-quality lead compounds across a broad chemical space.

## Supplementary Information

Below is the link to the electronic supplementary material.

Supplementary Material 1: Supporting information document (PDF): List of excluded R-groups; Structural alignment of human PDE9A structures; Results of unconstrained docking; Results of constrained docking; Thermodynamic cycle of RBFE; Validation of FEP+ and OpenFE (H pocket); Validation of NES with 4Y86; Validation of NES to various docking poses with 3JSW; Prediction of tautomer preferences; Validation of FEP+ and OpenFE (M pocket); Workflow of Step 1; NES calculations of Step 1; In-silico prediction of glucuronidation; and Visualization of H-Pocket Substituent Space

Supplementary Material 2: SMILES data of compounds (XLSX): Selected R-groups, Assessment of NES performance, R-groups of H pocket in Step 1, NES calculations in Step 2, and NES calculations in Step 3

## Data Availability

The Python scripts for compound generation, which build upon the methodology described in FEgrow (https://github.com/cole-group/FEgrow) and custom scripts for Non-equilibrium Switching (NES) calculations, are publicly available on GitHub (https://github.com/IkeguchiLab/InSilicoDrivenH2L). Molecular dynamics (MD) simulations were performed using the GROMACS 2024.1 software package (https://www.gromacs.org). Hybrid topologies for NES calculations were generated using pmx (https://github.com/deGrootLab/pmx). Additionally, the molecular simulation package licensed by Schrödinger, Inc. (https://www.schrodinger.com/) was used, and absorption, distribution, metabolism, excretion, and toxicity (ADMET) properties predictions were carried out using ADMET Predictor version 11.0, licensed by Simulations Plus, Inc. (https://www.simulations-plus.com/software/admetpredictor/).
