# Supplementary material for: In silico-driven protocol for hit-to-lead optimization: a case study on PDE9A inhibitors
Source: J Comput Aided Mol Des. 2025 Dec 19;40(1):24. doi: 10.1007/s10822-025-00729-7 (PMC12715073; doi:10.1007/s10822-025-00729-7)
Supplement: Supplementary file 1 — Supplementary Material 1: Supporting information document (PDF): List of excluded R-groups; Structural alignment of human PDE9A structures; Results of unconstrained docking; Results of constrained docking; Thermodynamic cycle of RBFE; Validation of FEP+ and OpenFE (H pocket); Validation of NES with 4Y86; Validation of NES to various docking poses with 3JSW; Prediction of tautomer preferences; Validation of FEP+ and OpenFE (M pocket); Workflow of Step 1; NES calculations of Step 1; In-silico prediction of glucuronidation; and Visualization of H-Pocket Substituent Space [file 10822_2025_729_MOESM1_ESM.pdf]

Supporting Information

In Silico-Driven Protocol for Hit-to-Lead Optimization: A Case Study on PDE9A Inhibitors

Hiroyuki Ogawa[†,‡], Masateru Ohta[§], Mitsunori Ikeguchi[*,†,§]

† Graduate School of Medical Life Science, Yokohama City University, 1-7-29 Suehiro-cho, Tsurumi-ku, Yokohama 230-0045, Japan

‡ Central Pharmaceutical Research Institute, Japan Tobacco Inc., 1-1, Murasaki-cho, Takatsuki, Osaka 569-1125, Japan

§ HPC- and AI-driven Drug Development Platform Division, Center for Computational Science, RIKEN 1-7-22, Suehiro-cho, Tsurumi-ku, Yokohama 230-0045, Japan

E-mail for correspondence: ike@yokohama-cu.ac.jp

Section S1. Basic Information

Table. S1 List of excluded R-groups

Substituents	SMARTS
ketone (C=O),<br>oxalic ester ((COO)2)	[#8]=[#6](-[#6])-[#6,#0]
nitro (NO2)	[#7](=[#8])-[#8]
ester (COOC)	[#8]=[#6,#0]-[#8]-[#6,#0]
N-oxide (NO),<br>amino alcohol (NO),<br>oxime (C=N-O)	[#8&A]-[#7&A] and [#8]-[#7&a]
thiourea (NC=SN)	[#6](=[#16])(-[#7])-[#7]
trithiocarbonate (S-C=S-S)	[#6](=[#16])(-[#16])-[#16]
thiocyanate (SCN)	[#6](-[#16])#[#7]
disulfide (S-S)	[#16]-[#16]
hydrazine (NN),<br>hydrazine carboxyl (N-N-C=O)	[#7&A]-[#7&A]
pyridinium ion	[#7+]1:[#6]:[#6]:[#6]:[#6]:[#6]:1
ammonium ion (N4+),<br>isocyano (NC),<br>imidazolium,<br>azide (N=N=N)	[#7+]
thioamide (N=C-S)	[#7]-[#6]=[#16]

acetal (OCO)	[#8]-[#6]-[#8]
sulfur monoxide (SO)	[#16&D3]=[#8]
thiosulfinate (SSO)	[#16]~[#16]~[#8]
guanidine (N-C=N-N)	[#6](-[#7])(=[#7])-[#7]
methane diamine (N-C-N)	[#7]-[#6](-[#7]) not ([#7]-[#6](-[#7])=[#8])
aminal (N-C-O)	[#8]-[#6]-[#7]
alkyne (CC)	[#6]#[#6]
thiol (SH)	[#16&H1]
aldehyde (CO),<br>formamide (N-C=O)	[#6&H1]=[#8]
thioether (C-S-C)	[#16&D2](-[#6])-[#6] and [#16&D2](-[#6])-[#0]
imidate (C=N-O)	[#6](=[#7])-[#8]
fluoro sulfone (SO2F)	[#16](=[#8])(=[#8])-[#9]
tetrahydro pyridine	[#7]1-[#6]-[#6]-[#6]-[#6]-[#6]=1
diphosphate (P-O-P)	[#15]-[#8]-[#15]
sulfonium (S+)	[#16+]
methylene bisphosphine (P-C-P)	[#15]-[#6]-[#15]
quinone	[#6]1:[#6]:[#6](:[#6]:[#6]:[#6]:1-[#8])-[#8]
o-fluoro pyridine	[#7]1:[#6](:[#6]:[#6]:[#6]:[#6]:1)-[#9]<br>not ([#7]1:[#6](:[#6]:[#6](:[#6]:[#6]:1)-[#9])=[#8]<br>and [#7]1(:[#6]:[#6]:[#6](:[#6]:[#6]:1)-[#9]):[#6,#7])
p-fluoro pyridine	[#6]1:[#6]:[#6](-[#9]):[#6]:[#6]:[#7]:1<br>not ([#6]1:[#6]:[#6](-[#9]):[#6]:[#6]:[#7]:1:[*])

isothiocyanate (N=C=S)	[#7]=[#6]=[#16]
heteroAr(N)-N	[#7&a]-[#7]
thiophosphine (PS)	[#15]~[#16]
tBoc (NCOOtBu)	[#7]-[#6](=[#8])-[#8]-[#6](-[#6])(-[#6])-[#6]
epoxy (-C-O-C-)	[#6]1-[#8]-[#6]-1
tosyl (tol-SO2O),<br>mesyl (Me-SO2O),<br>sulfonic acid (SO2H)	[#8]-[#16](=[#8])(=[#8])-[#6] and [#16](-[#8])(=[#8])=[#8]
diazene (N=N),<br>diazonium (NN)	[#7]=[#7]
nitroso (N=O)	[#7]=[#8]
thionitroso (N=S)	[#7]=[#16] not ([#7]=[#16]=[#8])
aziridine (-C-N-C-)	[#6]1-[#7]-[#6]-1
methylene sulfinyl (C=S)	[#6]=[#16]
peroxide (O-O)	[#8]-[#8]
phosphate (OPO(OEt)2)	[#8]-[#15](-[#8]-[#6])(=[#8])-[#8]-[#6]
alkene	[#6]=[#6]<br>not ([#7,#6,#8]1-[#6]-[#6]-[#6]=[#6]-[#6]-1<br>(not ([#6]=[#6]-[#6]=[#6] and [#6]=[#6]-[#6]-[#6]-[#6]=[#6]<br>and [#6](-[#6]=[#6])=[#8]))
amidine	[#7]=[#6]-[#7]
imine	[#7](=[#6])-[#0]<br>and [#6](=[#7])-[#0]<br>and [#6&A]=[#7]

	and [#6]=[#7]
cyanamide (N-CN)	[#7]-[#6]#[#7]
fluoro phosphine (PF)	[#15]-[#9]
phosphine monooxide (PO)	[#15]=[#8] not ([#15](=[#8])~[#8])
ethyl biphosphine (PCCP)	[#15]-[#6]-[#6]-[#15]
trifluoro sulfone (SO2CF3)	[#16](=[#8])(=[#8])-[#6](-[#9])(-[#9])-[#9]
fluoro triazine	[#6]1(:[#7]:[#6]:[#7]:[#6]:[#7]:1)-[#9]
sugar	[#8]1-[#6]-[#6](-[#6](-[#6](-[#6]-1)-[#8])-[#8])-[#8] and [#6]1-[#8]-[#6](-[#6]-[#6]-1-[#8])-[#6]-[#8] and [#6]1-[#8]-[#6](-[#6](-[#6]-1)-[#8])-[#6&D2]-[#8] and [#6]1-[#6]-[#6](-[#6](-[#6]-1)-[#8])-[#6&D2]-[#8] and [#6]1(-[#6]-[#6]-[#6](-[#6]-1-[#8])-[#8])-[#8] and [#8]1-[#6]-[#6]-[#6](-[#6](-[#6]-1-[#6&D2]-[#8])-[#8])-[#8])-[#8] and [#8]1-[#6]-[#6](-[#6]-[#6](-[#6]-1-[#6&D2]-[#8])-[#8])-[#8] and [#8]1-[#6]-[#6](-[#6](-[#6]-[#6]-1-[#6&D2]-[#8])-[#8])-[#8] and [#6]1-[#6]-[#6](-[#6](-[#6](-[#6]-1-[#6&D2]-[#8])-[#8])-[#8])-[#8]
amino thio ether(NS)	[#16&D2]-[#7]
imino sugar	[#6]1-[#7]-[#6](-[#6](-[#6]-1-[#8])-[#8])-[#6&D2]-[#8] and [#7]1-[#6]-[#6]-[#6](-[#6](-[#6]-1-[#6&D2]-[#8])-[#8])-[#8])-[#8]

	and [#7]1-[#6]-[#6](-[#6](-[#6](-[#6]-1)-[#8])-[#8])-[#8]
benzne-NO	[#6]1:[#6]2:[#6](:[#6]:[#6]:[#6]:1):[#8]:[#7]:2
benzene-cyclobutene	[#6]1:[#6]2:[#6](:[#6]:[#6]:[#6]:1)-[#6]-[#6]-2
ring open sugar	[#6](-[#8])-[#6](-[#6](-[#8])-[#6](-[#6]-[#8])-[#8])-[#8]
cyclobutene dione	[#6]1(:[#6](:[#6]:[#6]:1)=[#8])=[#8]
pyridine cyclopropane	[#6]1:[#6]2:[#6](:[#6]:[#7]:[#6]:1)-[#6]-2

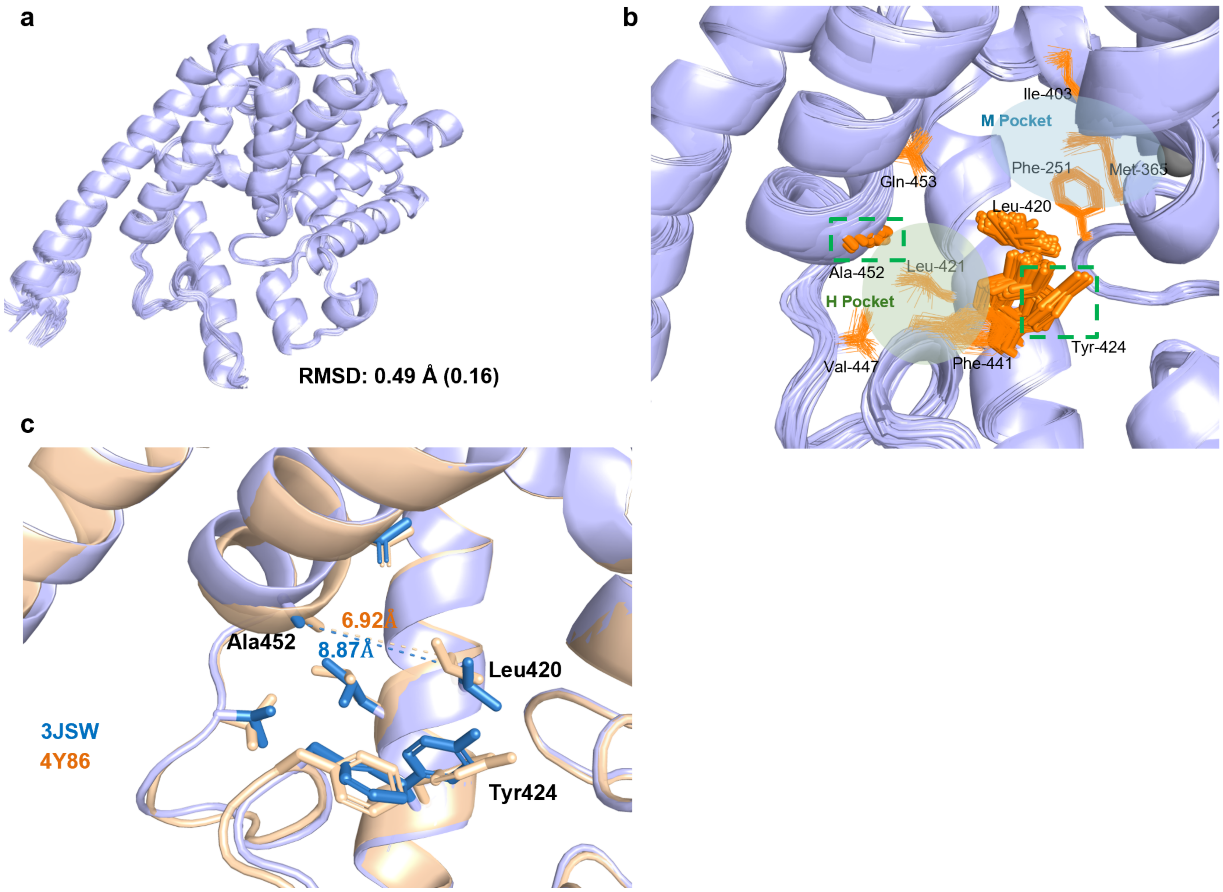

Fig. S1 Structural alignment of reported human PDE9A crystal structures. All available human PDE9A crystal structures (PDB IDs: 2HD1, 2YY2, 3DY8, 3DYL, 3DYN, 3DYQ, 3DYS, 3JSI, 3JSW, 3K3E, 3K3H, 3N3Z, 3QI3, 3QI4, 4E90, 4G2J, 4G2L, 4GH6, 4Y86, 4Y87, 4Y8C, 6A3N, 6LZZ, 7F0I, and 8BPY) were superimposed on chain A of 3JSW based on backbone atoms. (a) The overall backbone RMSD among all structures was 0.49 Å with a standard deviation of 0.16 Å. (b) Representative residues within the active site are shown. Tyr424 adopts an alternative conformation in three structures (4Y86 chain A, 4Y8C chain A, and 4Y8C chain B), and Ala452 exhibits poor overlap across structures. (c) Comparison between 3JSW and 4Y86. The Ala452 Cβ–Leu420 Cβ distance was 8.87 Å (ranked 41st among 50 chains) in 3JSW and 6.92 Å (ranked 3rd among 50 chains) in 4Y86. These two receptors represent one of the pairs showing the largest difference in H-pocket geometry among all PDE9A structures.

Section S2. Comparison of unconstrained and constrained docking

When performing *unconstrained* docking using two receptor structures, 3JSW and 4Y86, we observed that for some compounds, the docking poses differed substantially between the two receptor structures (Fig. S2). Particularly, some poses in both receptor structures failed to maintain the complementary hydrogen-bond interaction between Gln453 and the 3-cyanopyridone core, which is likely critical for the functional activity of PDE9A. In contrast, when *core-constrained* docking was performed, as described in the main text, comparable binding poses were obtained for both receptors, with the key Gln453–core hydrogen bond reliably maintained (Fig. S3). To minimize pose sensitivity to receptor conformation, we employed the *core-constrained* docking procedure to ensure preservation of this key interaction during H2L optimization.

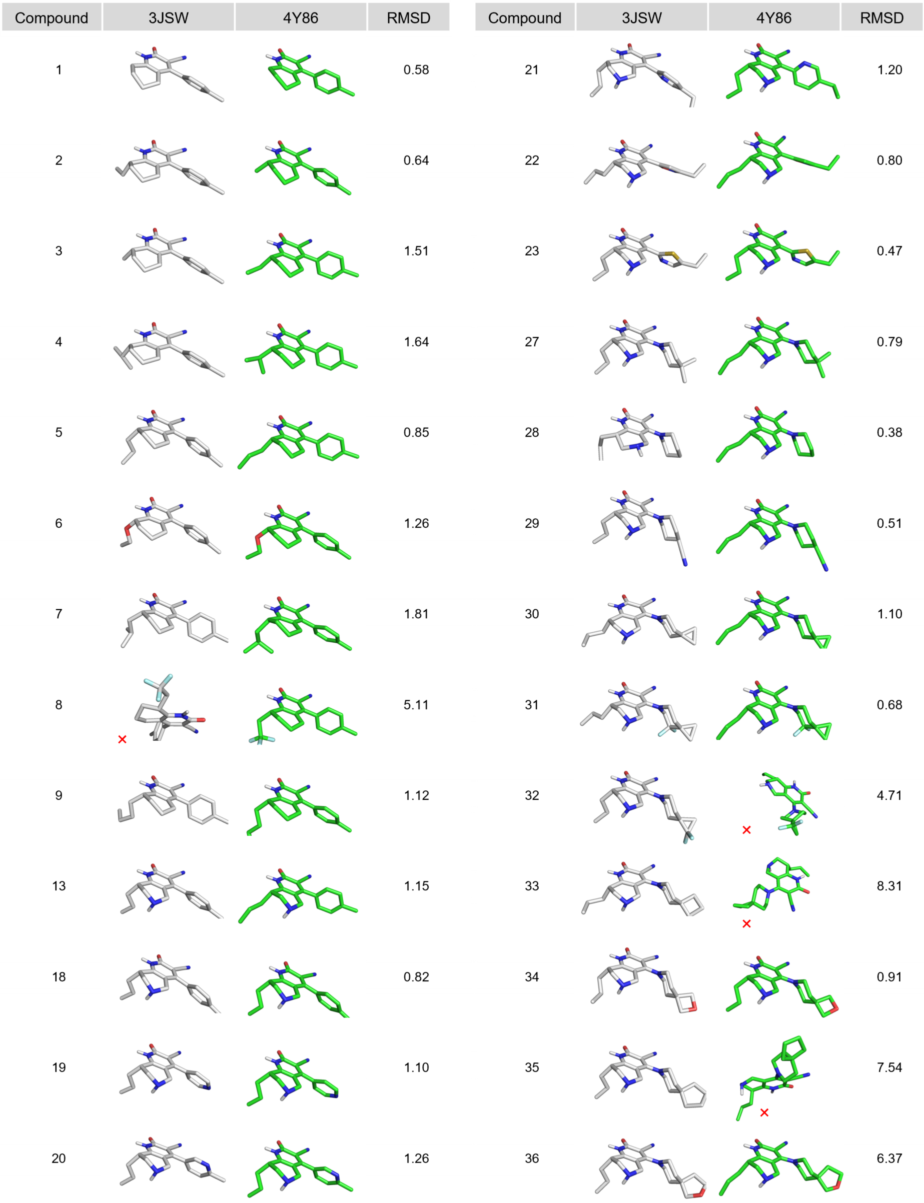

Compound	3JSW	4Y86	RMSD	Compound	3JSW	4Y86	RMSD
1			0.58	21			1.20
2			0.64	22			0.80
3			1.51	23			0.47
4			1.64	27			0.79
5			0.85	28			0.38
6			1.26	29			0.51
7			1.81	30			1.10
8	✕		5.11	31			0.68
9			1.12	32		✕	4.71
13			1.15	33		✕	8.31
18			0.82	34			0.91
19			1.10	35		✕	7.54
20			1.26	36			6.37

Fig. S2 Docking poses of literature-reported compounds obtained using the PDE9A receptor structures 3JSW and 4Y86 without applying core constraints. The protein backbones were aligned, and RMSD values between the corresponding ligand poses were calculated. For docking into 3JSW, Compound 8 failed to maintain the complementary hydrogen-bond interaction between Gln453 and the 3-cyanopyridone core. Similarly, for docking into 4Y86, Compounds 32, 33, and 35 did not maintain this key interaction.

Compound	3JSW	4Y86	RMSD	Compound	3JSW	4Y86	RMSD
1	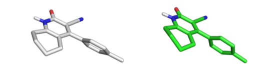		0.63	21	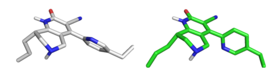		1.41
2	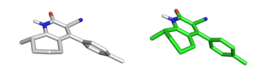		0.68	22	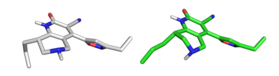		1.00
3	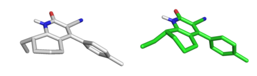		1.09	23	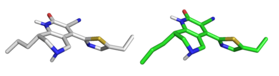		0.82
4	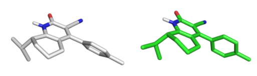		1.08	27	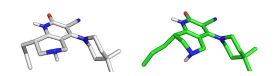		0.99
5	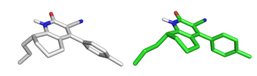		1.30	28	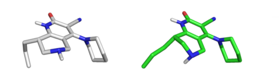		0.89
6	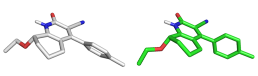		1.22	29	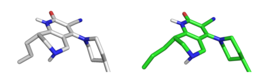		0.79
7	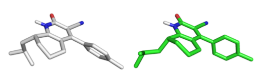		1.40	30	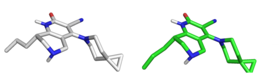		0.89
8	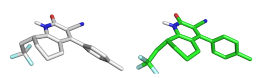		1.52	31	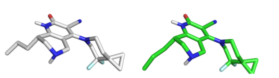		0.72
9	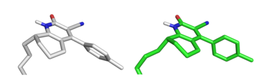		1.09	32	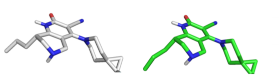		0.81
13	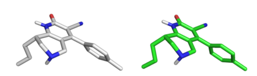		0.50	33	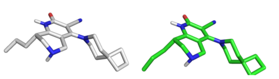		0.89
18	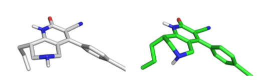		0.92	34	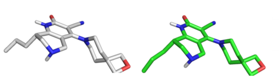		0.91
19	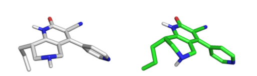		0.94	35	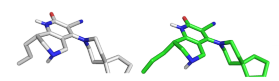		0.80
20	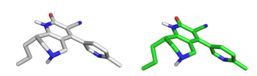		0.51	36	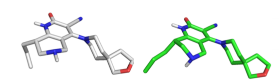		0.93

Fig. S3 Docking poses of literature-reported compounds obtained using the PDE9A receptor structures 3JSW and 4Y86 with the core constraint. The protein backbones were aligned, and RMSD values between the corresponding ligand poses were calculated.

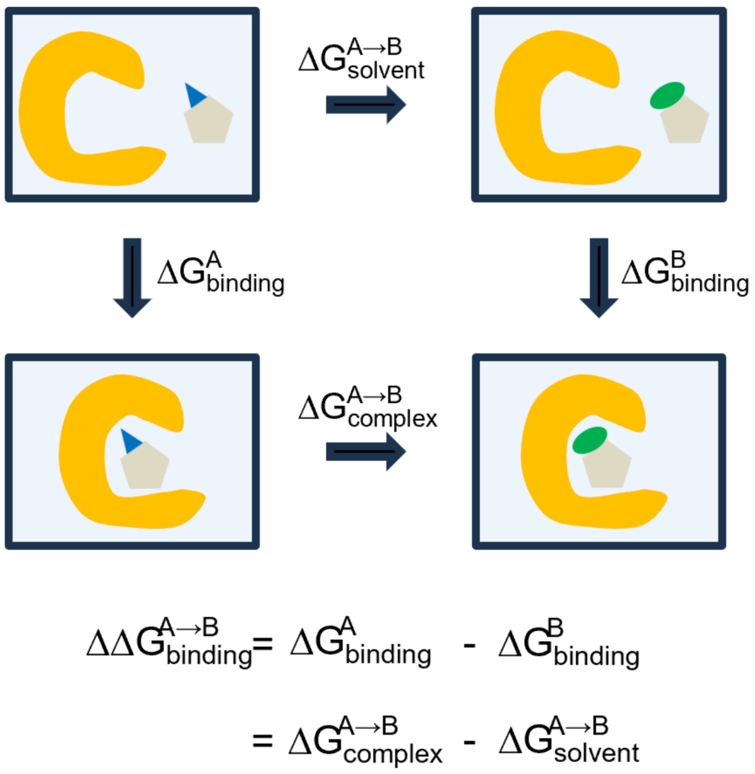

Fig. S4 Thermodynamic cycle used for the calculation of relative binding free energy (RBFE). The horizontal legs represent alchemical transformations between ligands in the bound (complex) and unbound (solvent) states, while the vertical legs correspond to physical binding processes. The RBFE (ΔΔG) was obtained by computing the free energy difference between the two horizontal transformations.

Supplementary Methods

Supplementary Method 1. FEP+ Calculations

Free Energy Perturbation (FEP) calculations were performed using the Schrödinger modeling suite (version 2024-3) [1] with the OPLS4 force field [2]. The replica exchange with solute tempering (REST) region was applied only to the heavy atoms of the ligand. Any missing force field parameters were derived using the Force Field Builder [3] by fitting to quantum mechanical (QM) calculations. Each FEP calculation employed 12 λ windows with 5 ns of production simulation per window, using default settings [4]. All calculations were repeated three times with different random seeds. Error bars in the figures represent the standard error of the ΔΔG values across the three runs.

Supplementary Method 2. OpenFreeEnergy (OpenFE) Calculations

Free Energy Perturbation (FEP) calculations were performed using OpenFreeEnergy (OpenFE) version 1.0.1 (https://github.com/OpenFreeEnergy/openfe). Ligands were parameterized with the Open Force Field 2.1.1 ("Sage") [5], and partial charges were assigned using the AM1-BCC method [6] The protein was parameterized using the Amber14SB force field.[7] Systems were solvated in explicit TIP3P water[8] and neutralized with counterions. Each FEP calculation used 11 λ windows with 5 ns of production simulation per window, using default settings. All calculations were repeated three times with different random seeds. Error bars in the figures represent the standard error of the ΔΔG values across the three runs.

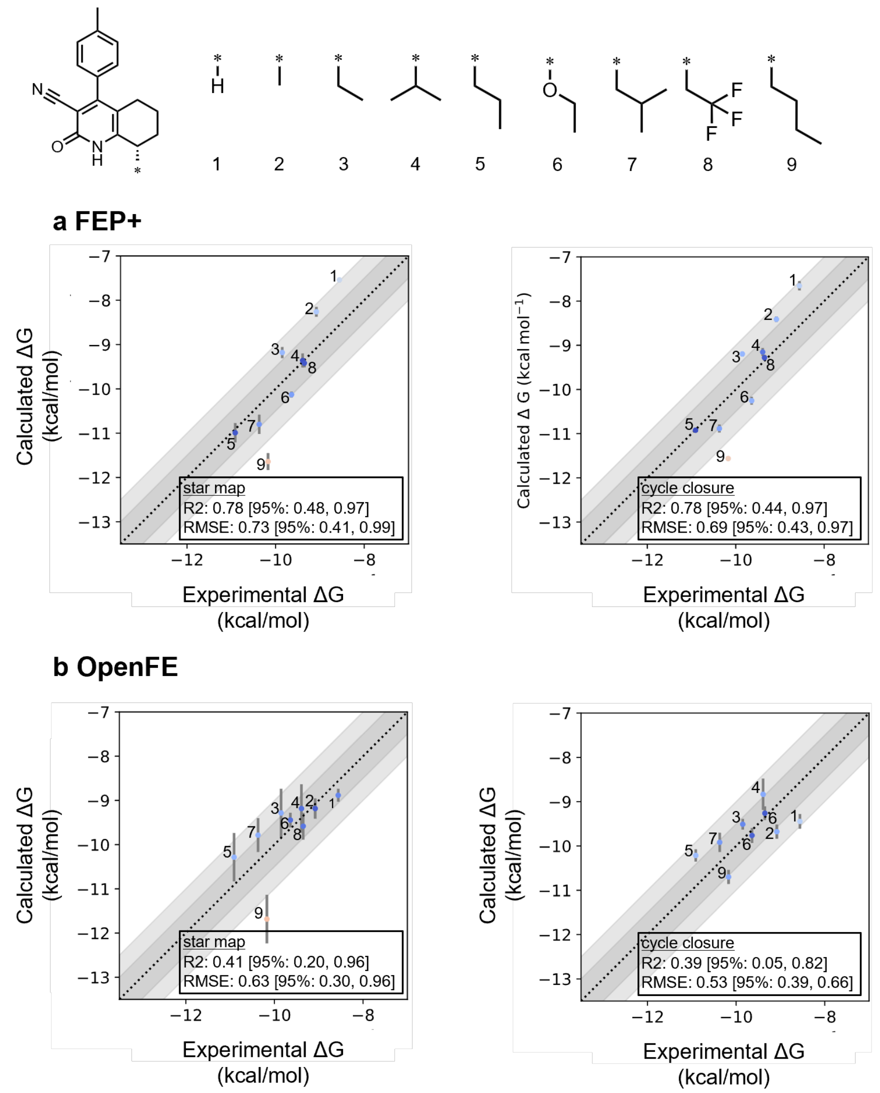

Fig. S5 FEP+ and OpenFreeEnergy (OpenFE) calculations were conducted using literature compounds for the H pocket. Compound 1 served as the reference in the star map. A perturbation map for cycle closure was generated using FEP+. Statistical evaluation was based on R^2 and RMSE values. Vertical error bars indicate the standard deviations of NES results, while the 95% confidence intervals for R^2 and RMSE were estimated by bootstrap resampling (n = 1000).

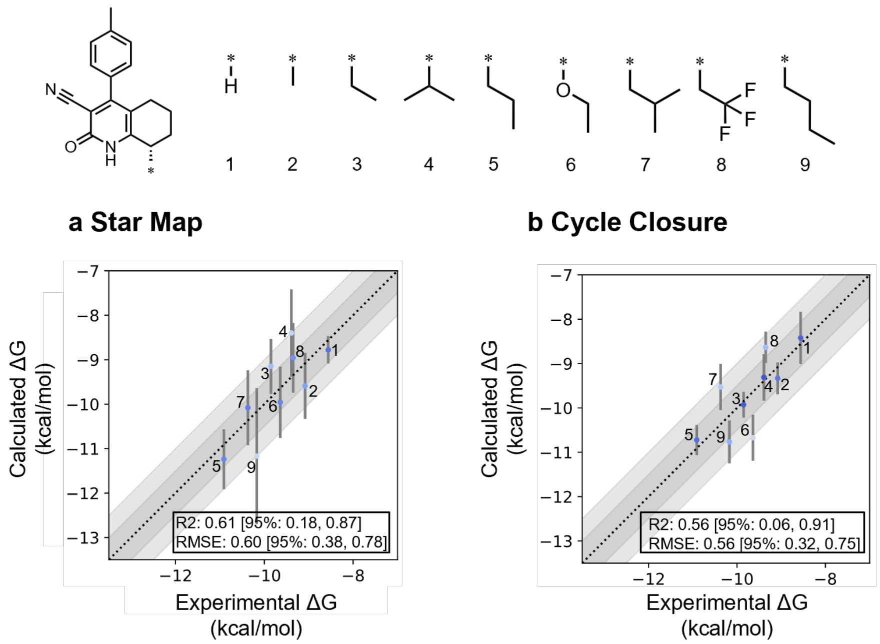

Fig. S6 NES calculations for the H pocket using literature compounds with 4Y86 structure. Compound 1 was used as the reference in the star map. The cycle-closure map was generated using FEP+, and accuracy was evaluated using R^2 and RMSE. The gray bands represent ± 0.5 and ± 1 kcal/mol from the ideal. Vertical error bars indicate the standard deviations of NES results, while the 95% confidence intervals for R^2 and RMSE were estimated by bootstrap resampling (n = 1000).

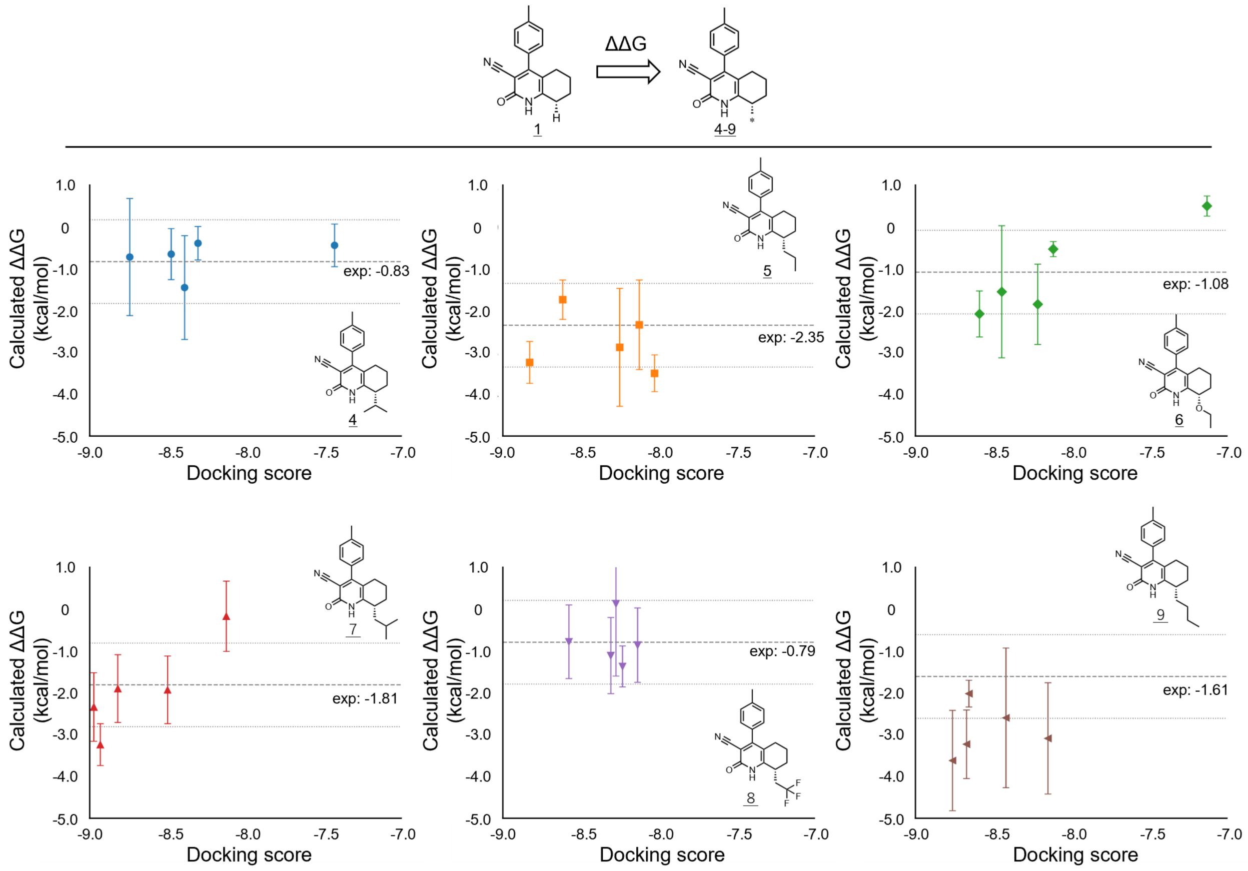

Fig. S7 To evaluate the sensitivity of NES calculations to docking poses for 3JSW, the five top-ranked docking poses were used for each of six compounds (4–9), with compound 1 serving as the reference. Dashed lines indicate the ±1 kcal/mol range from the experimental ΔΔG values, and vertical error bars represent the standard deviations of NES results.

Supplementary Method 3. Prediction of Tautomer Preferences

Tautomer preferences were predicted using the Jaguar QM Conformer and Tautomer Prediction tool (Schrödinger Suite 2024-3)[9] with default settings. For each compound, five poses were generated, and the lowest-energy tautomer was set as the reference (0 kcal/mol). Final relative energies (kcal/mol) were calculated relative to this reference. Compounds for which no tautomer could be generated were marked with "–". The Epik[10] state penalty was the value assigned to the generated tautomer in LigPrep.

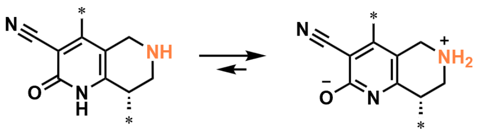

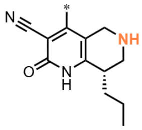

Compound	Structure	Jaguar Tautomer 1	Jaguar Tautomer 2	Epik (State Penalty) Tautomer 1	Epik (State Penalty) Tautomer 2
13	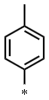	0	0.89	0.39	0.62
18	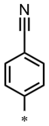	1.35	0	0.39	0.62
19	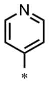	1.33	0	0.49	0.50
20	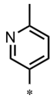	1.23	0	0.47	0.51
21	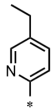	1.22	0	0.73	0.49

Compound	Structure	Jaguar Tautomer 1	Jaguar Tautomer 2	Epik (State Penalty) Tautomer 1	Epik (State Penalty) Tautomer 2
22	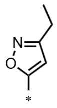	2.39	0	0.88	0.38
23	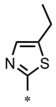	0.18	0	0.40	0.77
27	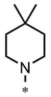	0	-	0.16	1.60
28	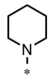	0	-	0.16	1.60
29	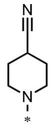	0	-	0.16	1.60
30	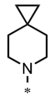	0	-	0.16	1.60

Fig. S8 Prediction of Tautomer Preference. Compounds 18–23 were found to favor Tautomer 2 based on the results of the Jaguar calculations. Additionally, these compounds exhibited only a small difference in Epik State Penalty between the two tautomers.

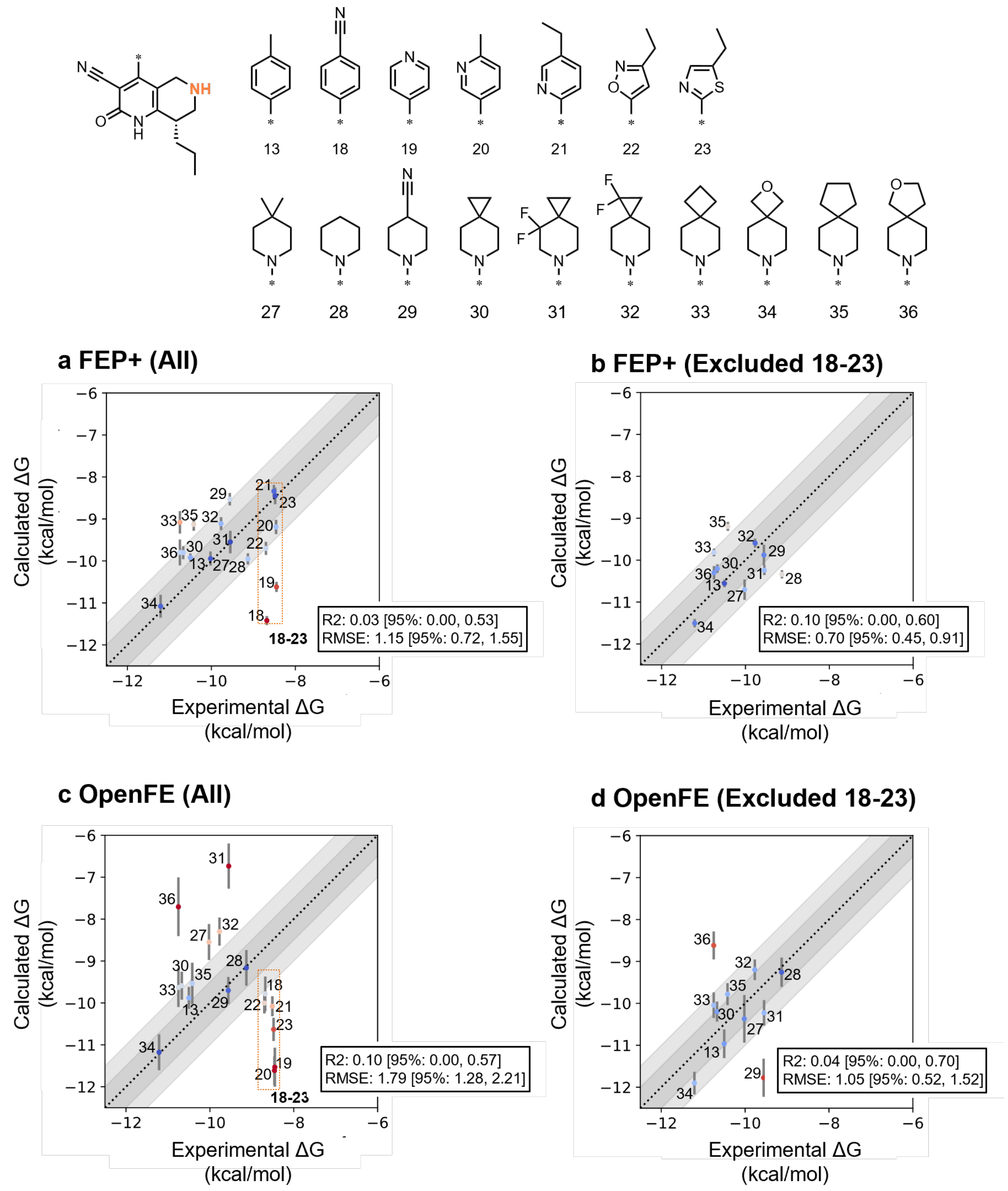

Fig. S9 FEP+ and OpenFreeEnergy (OpenFE) calculations were conducted using compounds from the literature for the M pocket. The perturbation map for the cycle closure was generated using FEP+. Two validation sets were examined: one that included all compounds and another that excluded Compounds 18–23. Statistical evaluation was based on R^2 and RMSE values. Vertical error bars indicate the standard deviations of NES results, while the 95% confidence intervals for R^2 and RMSE were estimated by bootstrap resampling (n = 1000).

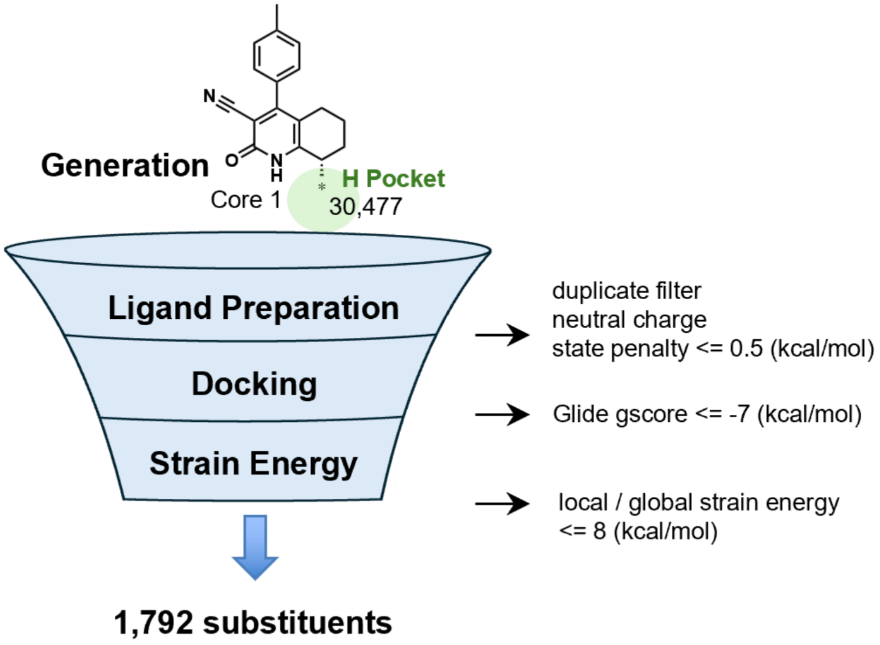

Fig. S10 Workflow of step 1 for selecting candidate substituents for the H pocket based on docking. A total of 1,792 substituents were selected.

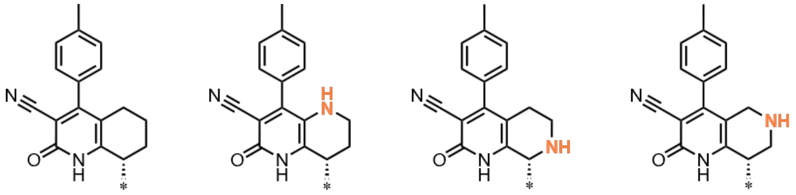

	*	ΔΔG (kcal/mol)
Core 1	H	0.0 ± 0.12
	Me	-0.58 ± 0.13
Core 2	H	0.79 ± 0.17
	Me	0.26 ± 0.16
Core 3	H	1.18 ± 0.14
	Me	0.76 ± 0.18
Core 4	H	1.22 ± 0.15
	Me	-1.47 ± 0.25

Fig. S11 NES calculations with full cycle closure were performed on representative compounds of each core, using Core 1 with hydrogen for the H pocket as the reference (ΔΔG = 0).

Section S3. In Silico Prediction of Glucuronidation Liability

Glucuronidation is a major Phase II metabolic pathway catalyzed by uridine 5′ -diphospho-glucuronosyltransferases (UGTs), which are primarily expressed in the liver, kidneys, and intestines. UGT enzymes conjugate glucuronic acid to nucleophilic functional groups, thereby increasing compound polarity and facilitating excretion. Among the UGT family, the UGT1 and UGT2 subfamilies, encompassing 22 known isoforms, are most relevant to drug metabolism. While glucuronidation generally facilitates drug clearance, acyl glucuronides derived from carboxylic acids can be reactive and potentially toxic, presenting a notable risk in drug development [11]. In the present study, the incorporation of a piperidine moiety into the M pocket was previously reported to reduce glucuronidation liability.

Several *in-silico* approaches for predicting glucuronidation potential have been reported [12, 13], and tools such as ADMET Predictor allow for the estimation of a compound's likelihood of serving as a substrate for major UGT isoforms. In this study, we employed ADMET Predictor version 11.0 (Simulations Plus, Inc.) to assess the glucuronidation liability of compounds across nine UGT isoforms. Among four compounds reported by Bayer, for which experimental metabolism data were available, only compound 27 was found to undergo primarily oxidative metabolism in rat hepatocytes, suggesting minimal glucuronidation. The prediction results for the four compounds are shown in Fig. S12a. Additionally, all 1,355 compounds evaluated by NES in the M-pocket optimization campaign were subjected to the same prediction. The results showed a broad distribution in predicted UGT liability (Fig. S12b), but no clear threshold could be defined based on literature or known compounds. These findings illustrate the difficulty of reliably assessing glucuronidation risk at the H2L stage using *in-silico* tools alone. While such predictions offer

valuable early-stage insights, robust evaluation of glucuronidation liability ultimately requires experimental validation.

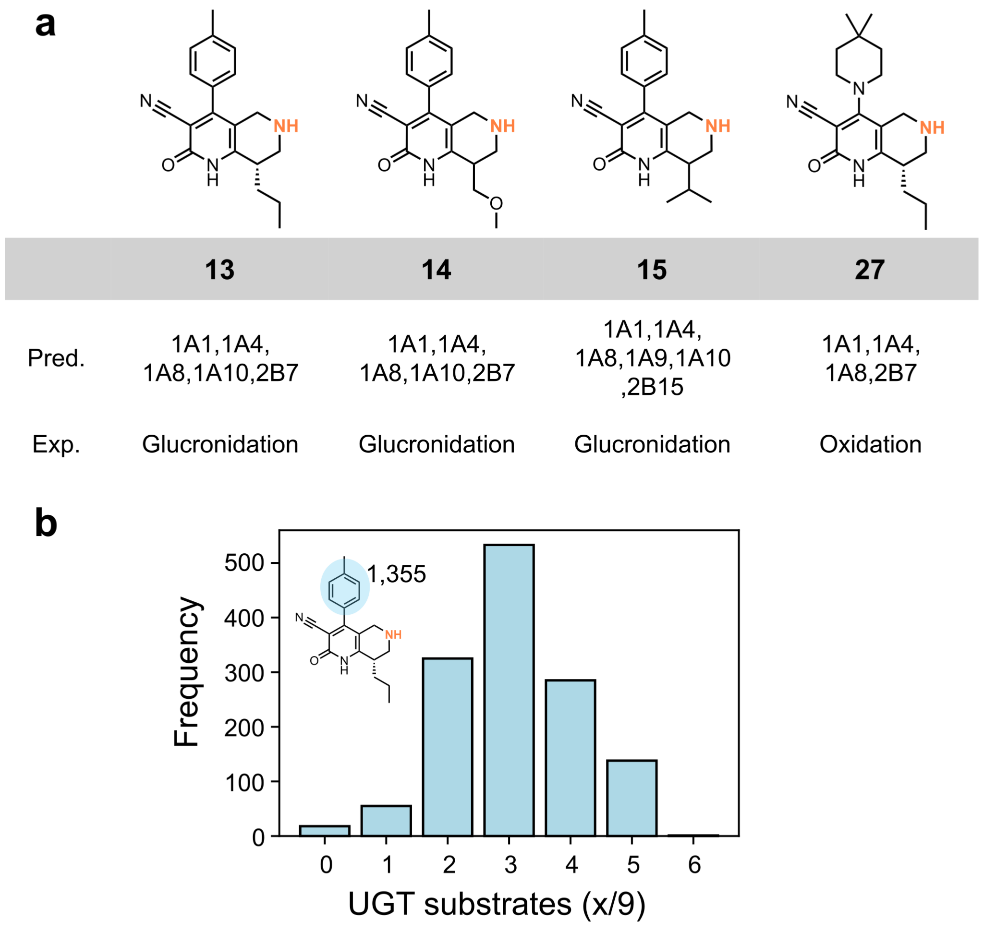

Fig. S12 *In-silico* prediction of glucuronidation liability. Binary classification was used to predict whether each compound acts as a substrate for nine UGT isoforms. (a) Predictions were performed for compounds with reported metabolites in rat hepatocytes. Compound 27 has been reported to undergo oxidative metabolism instead of glucuronidation. (b) Predictions were also performed on 1,355 compounds subjected to NES calculations. The histogram shows the number of UGT isoforms predicted to metabolize each compound.

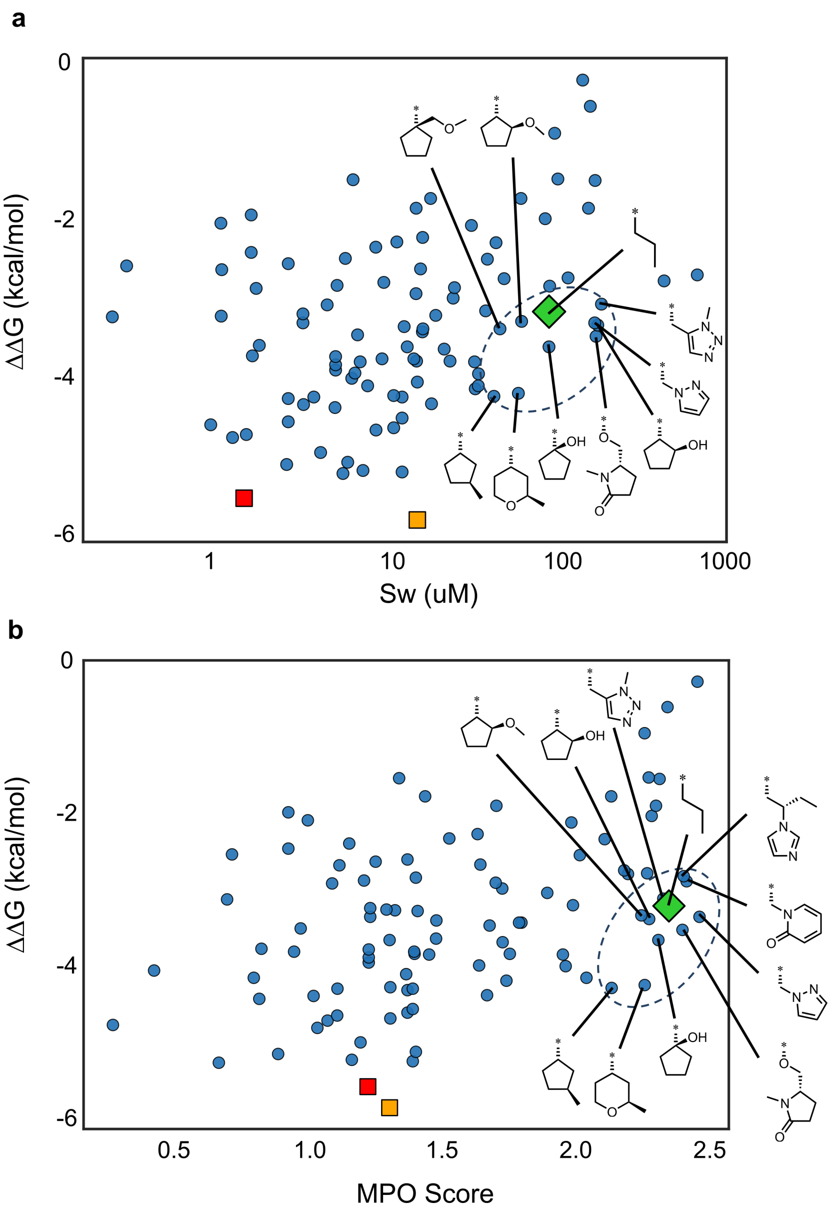

Fig. S13 Visualization of H-pocket Substituent Space using Predicted Affinity and ADME Profiles. (a) Scatter plot of predicted solubility versus binding affinity (ΔΔG). (b) Scatter plot of MPO score versus binding affinity (ΔΔG). Each plot represents the results of H-pocket optimization using a diverse set of substituents. Dashed circles indicate regions where a favorable balance between binding affinity and either solubility (a) or MPO score (b) is achieved. Representative substituents within these favorable regions are highlighted with chemical structures.

References

1. Schrödinger release 2024-3: FEP+. Schrödinger, LLC, New York, NY, 2024.

2. Lu C, Wu C, Ghoreishi D, Chen W, Wang L, Damm W, Ross GA, Dahlgren MK, Russell E, Von Bargen CD, Abel R, Friesner RA, Harder ED (2021) OPLS4: improving force field accuracy on challenging regimes of chemical space. J Chem Theory Comput 17:4291–4300. https://doi.org/10.1021/acs.jctc.1c00302

3. Schrödinger release 2024-3: Force Field Builder. Schrödinger, LLC, New York, NY, 2024.

4. Wang L, Wu Y, Deng Y, Kim B, Pierce L, Krilov G, Lupyan D, Robinson S, Dahlgren MK, Greenwood J, Romero DL, Masse C, Knight JL, Steinbrecher T, Beuming T, Damm W, Harder E, Sherman W, Brewer M, Wester R, Murcko M, Frye L, Farid R, Lin T, Mobley DL, Jorgensen WL, Berne BJ, Friesner RA, Abel R (2015) Accurate and reliable prediction of relative ligand binding potency in prospective drug discovery by way of a modern free-energy calculation protocol and force field. J Am Chem Soc 137:2695–2703. https://doi.org/10.1021/ja512751q

5. Boothroyd S, Behara PK, Madin OC, Hahn DF, Jang H, Gapsys V, Wagner JR, Horton JT, Dotson DL, Thompson MW, Maat J, Gokey T, Wang L-P, Cole DJ, Gilson MK, Chodera JD, Bayly CI, Shirts MR, Mobley DL (2023) Development and benchmarking of open force field 2.0.0: the Sage small molecule force field. J Chem Theory Comput 19:3251–3275. https://doi.org/10.1021/acs.jctc.3c00039

6. Jakalian A, Bush BL, Jack DB, Bayly CI (2000) Fast, efficient generation of high-quality atomic charges. AM1-BCC model: I. Method. J Comput Chem 21:132–146. https://doi.org/10.1002/(SICI)1096-987X(20000130)21:2<132::AID-JCC5>3.0.CO;2-P

7. Maier JA, Martinez C, Kasavajhala K, Wickstrom L, Hauser KE, Simmerling C (2015) ff14SB: improving the accuracy of protein side chain and backbone parameters from ff99SB. J Chem Theory Comput 11:3696–3713. https://doi.org/10.1021/acs.jctc.5b00255

8. Jorgensen WL, Chandrasekhar J, Madura JD, Impey RW, Klein ML (1983) Comparison of simple potential functions for simulating liquid water. J Chem Phys 79:926–935. https://doi.org/10.1063/1.445869

9. Schrödinger release 2024-3: Jaguar. Schrödinger, LLC, New York, NY, 2024.

10. Schrödinger release 2024-3: Epik. Schrödinger, LLC, New York, NY, 2024.

11. Shipkova M, Armstrong VW, Oellerich M, Wieland E (2003) Acyl glucuronide drug metabolites: toxicological and analytical implications. Ther Drug Monit 25:1. https://doi.org/10.1097/00007691-200302000-00001

12. Wu B, Wang X, Zhang S, Hu M (2012) Accurate prediction of glucuronidation of structurally diverse phenolics by human UGT1A9 using combined experimental and in silico approaches. Pharm Res 29:1544–1561. https://doi.org/10.1007/s11095-012-0666-z

13. Huang M, Lou C, Wu Z, Li W, Lee PW, Tang Y, Liu G (2022) In silico prediction of UGT-mediated metabolism in drug-like molecules via graph neural network. J Cheminformatics 14:46. https://doi.org/10.1186/s13321-022-00626-3
